# Spidroins and Silk Fibers of Aquatic Spiders

**DOI:** 10.1038/s41598-019-49587-y

**Published:** 2019-09-20

**Authors:** Sandra M. Correa-Garhwal, Thomas H. Clarke, Marc Janssen, Luc Crevecoeur, Bryce N. McQuillan, Angela H. Simpson, Cor J. Vink, Cheryl Y. Hayashi

**Affiliations:** 1 Department of Evolution, Ecology, and Organismal Biology, University of California, Riverside, CA 92591 USA; 2 J. Craig Venter Institute, Rockville, MD 28050 USA; 3 Meeuwen-Gruitrode, Limburg, 3670 Belgium; 4 Limburg Dome for Nature Study, Provincial Nature Center, Genk, 3600 Belgium; 5 Photographing Nature, Rotorua, New Zealand; 6 Canterbury Museum, Christchurch, 8013 New Zealand; 7 Division of Invertebrate Zoology and Sackler Institute for Comparative Genomics, American Museum of Natural History, New York, NY 10024 USA

**Keywords:** Evolutionary genetics, Transcriptomics

## Abstract

Spiders are commonly found in terrestrial environments and many rely heavily on their silks for fitness related tasks such as reproduction and dispersal. Although rare, a few species occupy aquatic or semi-aquatic habitats and for them, silk-related specializations are also essential to survive in aquatic environments. Most spider silks studied to date are from cob-web and orb-web weaving species, leaving the silks from many other terrestrial spiders as well as water-associated spiders largely undescribed. Here, we characterize silks from three Dictynoidea species: the aquatic spiders *Argyroneta aquatica* and *Desis marina* as well as the terrestrial *Badumna longinqua*. From silk gland RNA-Seq libraries, we report a total of 47 different homologs of the spidroin (spider fibroin) gene family. Some of these 47 spidroins correspond to known spidroin types (aciniform, ampullate, cribellar, pyriform, and tubuliform), while other spidroins represent novel branches of the spidroin gene family. We also report a hydrophobic amino acid motif (GV) that, to date, is found only in the spidroins of aquatic and semi-aquatic spiders. Comparison of spider silk sequences to the silks from other water-associated arthropods, shows that there is a diversity of strategies to function in aquatic environments.

## Introduction

Spiders use silk throughout their lives and most spider species produce multiple, functionally differentiated silks for a variety of essential purposes. The importance of silk to spiders is especially dramatic for water-associated spiders that rely on silk to survive immersion. Yet, the vast majority of silk molecular studies have been on terrestrial spiders, particularly cob-web and orb-web weavers (e.g.^1–4^). Spidroins, a contraction of “spider fibroins”^5^, are the dominant structural proteins of spider silks. Spidroins have several distinctive characteristics. The primary structure of a spidroin is mostly composed of a central repetitive region, consisting of repeating blocks of sequence that are typically enriched for the amino acids glycine, alanine, and serine^3,6,7^. For example, MaSp1 (major ampullate spidroin 1) from *Latrodectus hesperus*, the Western black widow, is dominated by short glycine-rich regions and poly-alanine blocks that form β-sheets. These β-sheets correspond to the crystalline domains that confer remarkable strength to dragline silk^8–11^. Furthermore, spidroins possess conserved amino (N) and carboxyl (C)-terminal domains flanking the central repetitive region and that play a key role in fiber formation^12–14^. Spidroin terminal regions are also important for annotation spidroins^3^.

**Figure 1:**
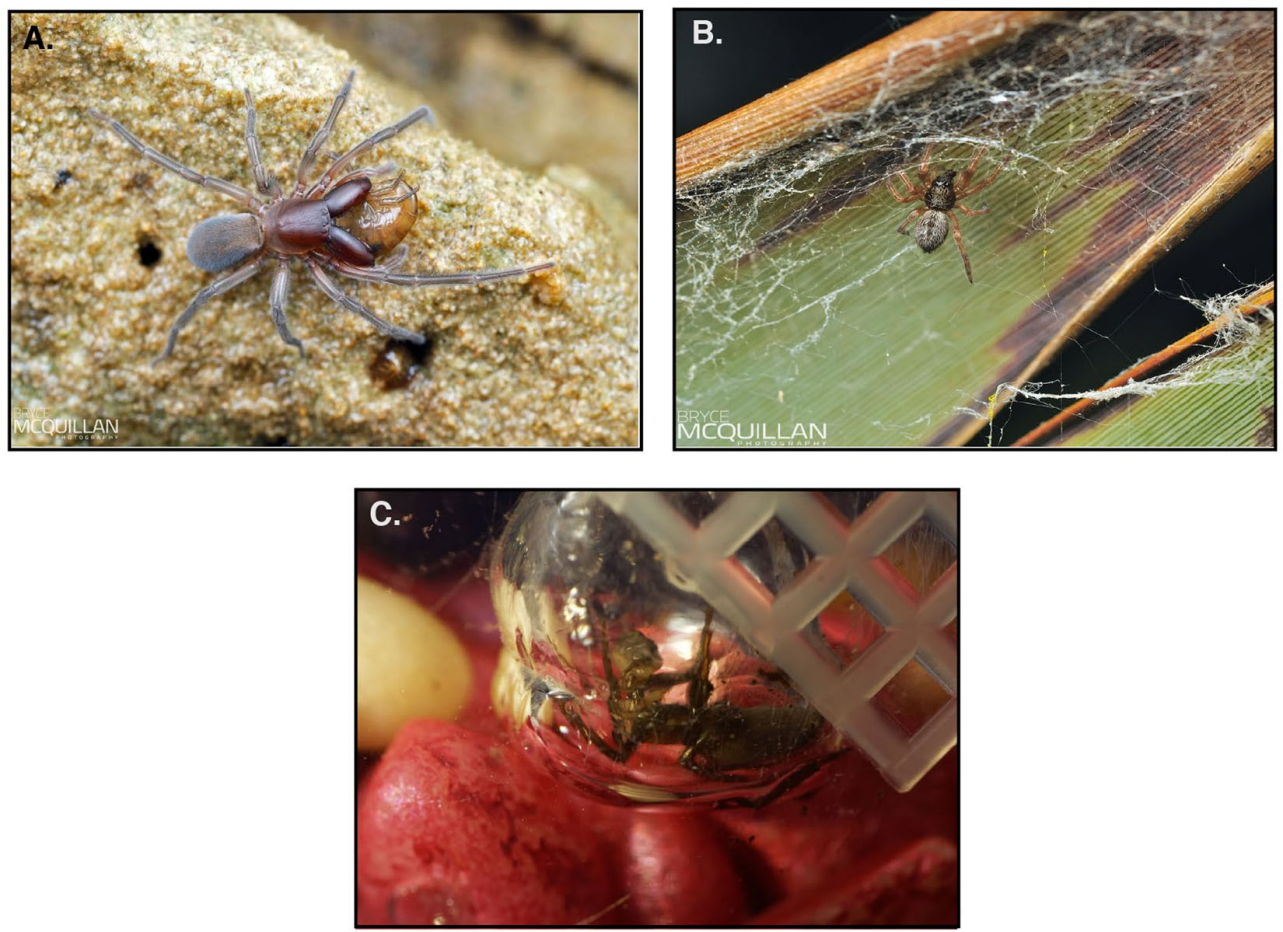
Focal spider species. Top view of *Desis marina* (Desidae) on sandstone at katikati, New Zealand (**A**). Top view of *Badumna longinqua* (Desidae) in its web (**B**). Side view of *Argyroneta aquatica* (Dictynidae) ventral side up inside its diving bell with a prey attached to its mouthparts (chelicerae) (**C**).

To date, the silks of spiders from diverse habitats, especially aquatic environments, are poorly characterized. However, such studies are needed to address questions regarding the relationship between silk specialization and ecology. Here, we compare spidroins, repeat sequence composition, and silk gene expression among spiders within the superfamily Dictynoidea, which has species with diverse lifestyles. Specifically, we focus on three spiders that use silk in three different environments: freshwater, marine, and non-aquatic^15^ (Fig. 1; *Argyroneta aquatica, Desis marina*, and *Badumna longinqua*).

Within Dictynoidea, *D. marina* and *B. longinqua*, members of the family Desidae, inhabit contrasting environments^16–18^. The medium-sized, ecribellate (lacking a cribellum due to secondary loss) spider *D. marina* lives within cavities in kelp holdfasts or holes in rocks along the shorelines of New Zealand^19–21^ (Fig. 1A). *Desis marina* spiders are not known to make prey-catching webs; instead, they catch intertidal amphipods by ambush^21^. Yet, silk is essential to these spiders for the construction of silk-lined retreats within kelp or rock cavities that protect them from tides and water pressure^22^. *Desis marina* spiders can remain submerged for up to 19 days by trapping air in their silken retreat, coupled with their lower respiration rate^19^.

Unlike the aquatic *D. marina*, *B. longinqua* is a medium-sized cribellate (possessing a cribellum) spider commonly found around buildings and small bushes in coastal urban and suburban areas^23^. *Badunma longinqua* spiders build lattice-like sheet webs that extend from tubular retreats in crevices such as dense foliage and small openings^23,24^ (Fig. 1B). The web contains cribellar silk, a dry adhesive matrix composed of small fibrils, which emerge from the cribellum, a plate-like spinning organ, and are combed by the spider as they are drawn out from the cribellar spigots^25–28^. Because *B. longinqua* and *D. marina* are in the same family, yet one is terrestrial and the other is water-associated, they serve as exemplars to investigate the role of spider silks in relation to their environment.

While *D. marina* is found in marine habitats, *A. aquatica* (Dictynidae, Dictynoidea), our third focal species, is found in freshwater ponds and lakes in Northern Europe, spending all of its life underwater^20^. Like *D. marina*, *A. aquatica* is ecribellate. *Argyroneta aquatica* spiders build an underwater domed-shaped sheet-web, called a diving-bell, which serves as an air reservoir^29,30^ (Fig. 1C). The spider transports air from above the water’s surface down to the diving-bell using an air bubble kept in place by abdominal hydrophobic hairs, referred to as a plastron^31,32^. While the *A. aquatica* underwater web can function as a physical gill, exchanging dissolved oxygen from water, periodic air renewal is still needed at long time intervals to avoid collapse of the web^30,33^.

We can predict the type of spidroin genes expressed in the focal species by the type of silk spigots that are present. Spidroins are secreted by abdominal silk glands, which are connected to spigots located on the spinnerets from where silk fibers are drawn. Silk spigots can be morphologically differentiated based on size and location. For example, major ampullate fibers are assembled from major ampullate spidroins produced in major ampullate glands connected to major ampullate silk spigots^34,35^. Therefore the silk spigot complex on a spider’s spinnerets can be an indicator of the silk types and underlying spidroins that the spiders produce. Morphological studies of *A. aquatica, B. longinqua*, and *D. marina* have identified silk spigots presumed to be connected to minor ampullate, major ampullate, pyriform, aciniform, and tubuliform silk glands^35,36^. Given that spidroins are named after the silk gland type in which they were first identified, we anticipate identifying the following spidroin genes in the three species: *MiSp* (an abbreviation of “minor ampullate spidroin”), *MaSp* (major ampullate spidroin), *PySp* (pyriform spidroin), *AcSp* (aciniform spidroin), and *TuSp* (tubuliform spidroin). In regard to *TuSp*, we expect expression limited to females as only females have tubuliform (egg case) spigots.

Although the three species have several silk spigots in common, they also have some differences. As a cribellate spider, *B. longinqua* has cribellar spigots on the cribellum that produce cribellar silk. *Badumna longinqua* also has an additional triad of neighboring spigots that are connected to uncharacterized silk glands. Each triad is composed of a large spigot called the “modified spigot” and two smaller, paracribellar spigots^35^. Similarly, *D. marina* also has a pair of “modified spigots” connected to unidentified silk glands^35^. Thus, we expect to identify *CrSp* (cribellar spidroin) in *B. longinqua* and new spidroin types in *B. longinqua* and *D. marina* that are associated with the uncharacterized silk glands connected to the modified and paracribellar spigots.

*Badumna longinqua* and *D. marina* are in the same family (Desidae) and are therefore more closely related to each other than either is to *A. aquatica*, which is in the family Dictynidae. Because they are in the same family, we expect *B. longinqua* and *D. marina* to have a similar complement of spidroin genes. Alternatively, if environment is a major selective force shaping spidroin sequences, spidroins from the water-associated spiders *A. aquatica* and *D. marina* could share more similarities. Furthermore, *A. aquatica* and *D. marina* spidroins may share similarities with those of another semi-aquatic spider, *Dolomedes triton*^37^ (Pisauridae).

## Results and Discussion

### AcSp, TuSp, and PySp sequences are conserved in the focal species

**Figure 2:**
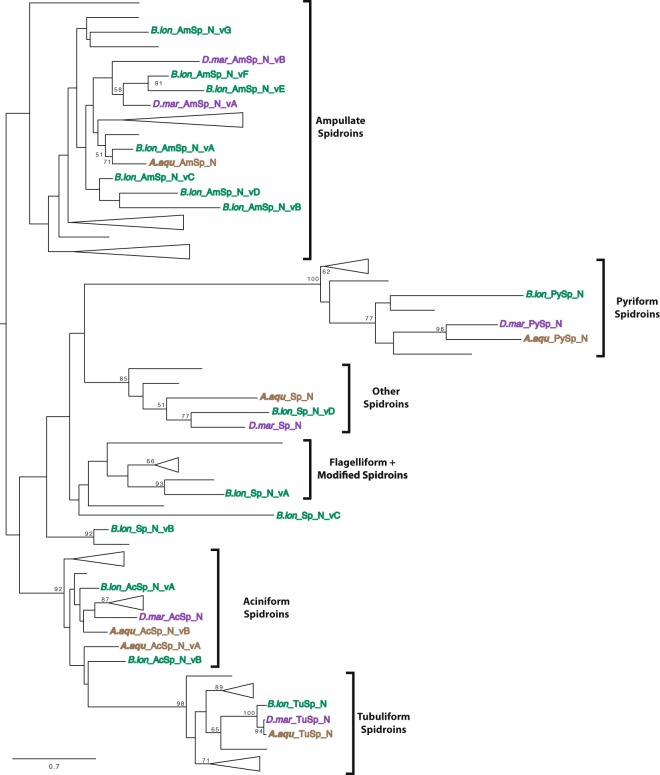
Phylogeny of spidroin N-terminal regions. *Argyroneta aquatica*, *Badumna longinqua*, and *Desis marina* spidroin paralogs highlighted in brown, green, and purple respectively. Tree is rooted with the *Bothriocyrtum californicum* fibroin 1 (not shown). Spidroin names are abbreviated as in Table S1. Bootstrap support percentages >50% are shown. The scale bar indicates 0.7 substitutions per site. See Supplementary Fig. S7 for complete tree with all sequence names.

**Figure 3:**
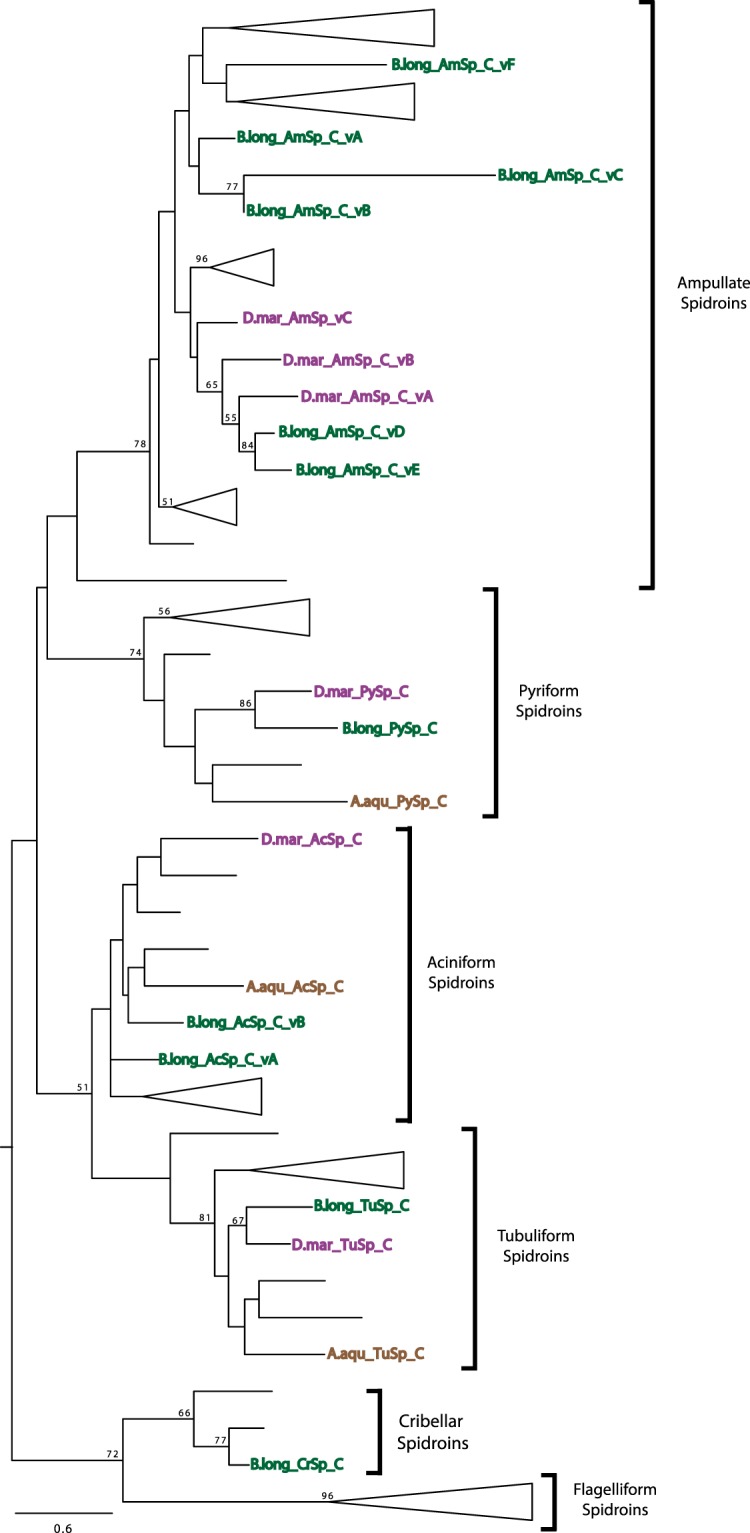
Phylogeny of spidroin C-terminal regions. Sequence names highlighted as in Fig. 2. Tree is rooted with the *Bothriocyrtum californicum* fibroin 1 (not shown). Spidroin names are abbreviated as in Table S1. Bootstrap support percentages >50% are shown. The scale bar indicates 0.6 substitutions per site. See Supplementary Fig. S8 for complete tree with all sequence names.

We identified nine, 26, and 12 spidroin contigs from the transcriptome assemblies of *A. aquatica, B. longinqua*, and *D. marina*, respectively (Supplementary Table S1). All 47 of these spidroin contigs were partial length transcripts containing sequence for either the N- or C-terminal encoding region. Most of the spidroin contigs (44 of 47) included adjacent repetitive sequence, and of these, 21 had significant sequence similarity to published repeat units of AcSp (used in prey-wrapping), TuSp (egg-case construction), or PySp (attaching silk fibers to substrates or to each other; Supplementary Fig. S1). Phylogenetic analyses of the terminal encoding regions recovered clades for AcSp, TuSp, and PySp spidroins (Figs 2 and 3). These clades are groupings of AcSp, TuSp, and PySp sequences of the focal species with the same spidroin types from the comparison species included in the analyses. As with previous hypotheses of spidroin evolution (e.g.^7,38–41^), a sister relationship of TuSp and AcSp clades was recovered in maximum likelihood analysis of the C-terminal encoding region (Fig. 3; 51% bootstrap support - BT). This result contrasted with the more weakly supported relationship of tubuliform spidroins nested within aciniform sequences that was recovered in the N-terminal domain analysis (Fig. 2; 17% BT).

The AcSp protein sequences have repeat units that are long (ranging from 173–206 aa) and there appear to be at least two loci each in *B. longinqua* and *A. aquatica*. Two loci for *A. aquatica* AcSp is consistent with previously published *A. aquatica* silk genes^42^. Dictynoid TuSp sequences also have long repeat units (ranging from 190–196 aa) and share the conserved amino acid sequence motifs: poly-serine, short runs (no more than four residues) of poly-alanine, and poly-threonine. The sequences are described in detail in Supplementary Text.

Pyriform silk, a composite of PySp-based fibers and a cement coating, is used to adhere a variety of silk fibers to a substrate or to each other^43–45^. Similar to AcSp and TuSp, PySp spidroins also show conservation of repeat length and composition with previously described PySp sequences^46–49^. Although a full repeat unit was not obtained for *D. marina*, *A. aquatica* and *B. longinqua* contigs did include complete PySp repeat units (Supplementary Fig. S1D). PySp repeat units of *A. aquatica* (207 aa) and *B. longinqua* (198 aa) are very similar to each other, sharing 80% amino acid identity. As with PySp from cob-web and orb-web weaving spiders, *A. aquatica* and *B. longinqua* PySp repeats are rich in serine and glutamine. They also share a proline-rich motif (boxed, Supplementary Fig. S1D) with repeats of PySp1 from orb-web weaving spiders, PySp2 from the cob-web weaver *Parasteatoda tepidariorum*, the fishing spider *D. triton* PySp, and PySp from the cribellate spiders *Tengella perfuga* and *Stegodyphus mimosarum*^46–48^. The *B. longinqua* proline-rich motif has the same length as that of the orb-web weaver *Argiope argentata* (36 aa). In orb-web weaving spiders, the proline-rich motif is thought to produce a random coil configuration that promotes elastomeric properties in pyriform silk^48^. In contrast to these two terrestrial spiders, the fully aquatic spider *A. aquatica* has a proline-rich motif that is the same length as that of the semi-aquatic spider *D. triton* PySp (28 aa). Overall similarities in repeat unit composition and length of PySp-specific motifs suggests similar selective pressures acting on PySp from terrestrial and aquatic spiders. Chemical composition and nano-structure studies of pyriform silk are needed to understand the mechanism of anchoring silk fibers to wet surfaces, and whether there is a functional significance to the water-associated PySp repeats having a shorter proline-rich motif.

While our sequences are partial length spidroin contigs, the portion of the repetitive region immediately adjacent to the N- and C- terminal region of each *B. longinqua* AcSp variant, as well as AcSp, TuSp, and PySp variants, respectively, from *D. marina*, were found to be nearly identical, suggesting that each pairing represents two ends of the same locus (Supplementary Fig. S2). This is the more parsimonious, conservative interpretation. It is also possible, however, that the similarity between the N- and C-terminal region transcripts may represent the ends of different gene copies with similar functions.

### Ampullate spidroin repeat sequences are diverse in the focal species

Many spiders use major and minor ampullate silk gland fibers as draglines and for the construction of prey-capture webs. Based on the presence of major and minor ampullate spigots on their spinnerets^35,36^, *A. aquatica, B. longinqua*, and *D. marina* were expected to express silk genes associated with major and minor ampullate silk glands. A total of 19 spidroin contigs were identified with similarities to described major (MaSp) and minor (MiSp) ampullate spidroins such as the repetitive region being mainly composed of alanine and glycine residues (Supplementary Fig. S4). Phylogenetic analyses show that these 19 sequences cluster with various MiSp and MaSp (e.g., MaSp1, MaSp2, MaSp3) sequences from other species in a large spidroin clade named Ampullate Spidroins (Figs 2 and 3). Because we could not definitively categorize these transcripts based on their repetitive region as either MaSp or MiSp, we annotated them with the neutral name “AmSp”, a contraction of “Ampullate Spidroins” conforming to the nomenclature of Collin *et al*.^50^. Each AmSp sequence name was followed by an “N” or “C” indicating if the transcript contains an amino or carboxyl-terminal region, and different AmSp paralogs are distinguish by a variant (v) letter.

We found that *B. longinqua* has more AmSp variants (six) compared to *A. aquatica* and *D. marina*, which appear to have only one or two variants, respectively (Supplementary Fig. S4). Regardless of species, all AmSp repeat regions have a predominance of glycine, alanine, and serine residues (Supplementary Fig. S4). These amino acids appear in a variety of short sequence motifs. For example, *B. longinqua* AmSp repeat regions have poly-glycine, poly-alanine, and poly-serine motifs (e.g., GGGG, AAAAA, SSS, respectively). Additionally, the *B. longinqua* AmSp variants differ in their proportion of glycine-alanine motifs (40% of *B. lon*_AmSp_N_vA vs. 24% of *B. lon*_AmSp_C_vC; Supplementary Fig. S4). While the AmSp sequences vary extensively within and across species, we found one motif, GGYGQ, to be common in the repeats of *A. aquatica* and *B. longinqua* (shaded, Supplementary Fig. S4).

Terrestrial spider webs are susceptible to changes in humidity. Under high humidity, the major ampullate silk of some species has been observed to supercontract^51–54^. Supercontraction, the sudden reduction in silk fiber length when wetted with water, is due to the disruption of hydrogen bonds between proteins that allows for re-orientation and coiling of silk molecules^55–57^. Major ampullate sequence elements from cob-web and orb-web weaving spiders such as GPGXX (X is one of a small subset of amino acids) and YGGLGS(N)QGAGR amino acid motifs are believed to be associated with supercontraction^52,58^. Because silks from *A. aquatica* and *D. marina* are in constant contact with water, we would expect ampullate sequences to have a shortage of these supercontraction elements as supercontraction would not be beneficial in aquatic habitats. Consistent with this prediction, AmSp sequences from *A. aquatica* and *D. marina* spiders were found to lack the amino acid motifs associated with supercontraction.

### Putative cribellar and modified spigot silk-specific spidroins in *B. longinqua*

Cribellate spiders use cribellar silk in their prey-capture web as a dry adhesive to secure freshly caught insects to their webs. A recent study of silks from the cribellate spider *T. perfuga* identified a putative spidroin gene associated with cribellar silk, called *CrSp* (Cribellar Spidroin^59^). In maximum likelihood analysis of the C- terminal encoding regions, we found a *B. longinqua* transcript that grouped with *T. perfuga* CrSp and a *S. mimosarum* spidroin with moderate support (Fig. 3; 67% BT). Furthermore, comparison of the repetitive regions of the *B. longinqua* transcript to those of *T. perfuga* and *S. mimosarum* shows that they share a well-conserved repeat unit (Supplementary Fig. S3; 51% aa identity). Thus, we annotated this *B. longinqua* transcript as Cribellar Spidroin (*B. lon*_C_CrSp; Supplementary Table S1).

Another transcript containing the N- terminal encoding region also showed affinities to spidroins from cribellate spiders. The transcript, *B. lon*_Sp_N_vA, grouped with a *T. perfuga* spidroin (*T. per*_Sp_N 93% BT; Fig. 2). The repetitive sequence of *B. lon*_Sp_N_vA has high amounts of glycine mainly organized in couplets (GX, with X representing a subset of polar amino acids), similar to the repetitive sequence of *T. per*_Sp_N. It has been hypothesized that *T. per*_Sp_N is associated with the modified/ pseudoflagelliform silk glands^59^, based on homology of *T. perfuga* modified spigots to the modified/pseudoflagelliform silk spigots in other cribellate species^60^. Modified spigots have also been documented for *B. longinqua*^35^. This morphological similarity, along with the grouping in the gene tree and shared repetitive sequence attributes, provide multiple lines of evidence that *B. lon*_Sp_N_vA and *T. per*_Sp_N represent the same spidroin type and are produced in the modified spigot glands.

### Evidence for additional spidroin types in the focal species

Five transcripts contain spidroin N-terminal encoding regions but do not have similarities in the repetitive region to any known spidroin types. Blast searches to NCBI nr and to our protein database (Supplementary Table S4) showed these sequences aligned to the N- terminal region of multiple spidroin types, confirming their classification as spidroins, but their repetitive regions were highly divergent from known spidroin repeats. Thus, we named each of them with only “Sp” for “Spidroin” and no indicator of type, such as the “Am,” “Cr,” “Py,” or “Tu” of AmSp, CrSp, PySp, and TuSp following the nomenclature of previous works with undetermined spidroin types (e.g.^1,37,59,61^). Maximum likelihood analyses shows one of the Sp sequences (*B. lon*_Sp_N_vB) groups with a *S. mimosarum* spidroin (*S. mim*_Sp2b) and their clade is positioned outside a diverse clade that includes pyriform, flagelliform, and cribellar spidroins (Fig. 2). The repetitive regions of *B. lon*_Sp_N_vB and *S. mim*_Sp2b also share 53% amino acid similarity, further support that they represent the same spidroin type (Supplementary Fig. S5).

Another novel spidroin, *B. lon*_Sp_N_vC, is sister to a clade containing cribellar and flagelliform spidroins (Fig. 2). The repetitive region of this spidroin is high in the amino acids glycine (36%) and serine (21%) that are largely present in glycine-serine couplets. However, glycine-serine couplets are not present in cribellar or flagelliform spidroins^62^ (Supplementary Fig. S3). Given its phylogenetic placement and distinctive repeat region characteristics, we were unable to associate *B. lon*_Sp_N_vC with any known spidroin type.

**Figure 4:**
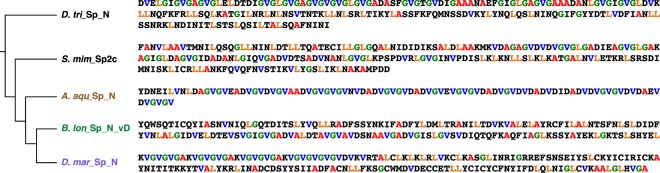
Comparison of exemplar repeats of sequences from the “Other Spidroins” clade shown in Fig. 2. Names for Sp spidroins from *Argyroneta aquatica, Badumna longinqua*, *Desis marina*, *Stegodyphus mimosarum*, and *Dolomedes triton* abbreviated as in Supplementary Tables S1 and S5. Abundant amino acids colored in red (alanine), blue (valine), green (glycine), and orange (leucine).

The last three novel spidroin transcripts, one from each focal species, encode proteins with similar N-terminal regions to each other but different repeat units. The transcripts *A. aqu_*Sp_N, *B. lon*_Sp_N_vD, and *D. mar*_Sp_N form a well-supported clade with other spidroins from *D. triton* and *S. mimosarum* (*D. tri*_Sp_N and *S.mim*_Sp2c; Fig. 2; 85% BT). Given that members of this clade have an unclear relationship to known spidroin types and do not have motifs that could affiliate them with previously known spidroin types, we refer to this clade as “Other Spidroins” (Figs 2 and 4). However, the spidroin repetitive regions from *A. aquatica*, *D. triton*, and *D. marina* spiders, which are aquatic or semi-aquatic species, do share some similarities. The repetitive region sequences in *A. aqu_*Sp_N, *D. mar*_Sp_N, and *D. tri*_Sp_N have repeated couplets of the hydrophobic amino acids glycine and valine (Fig. 4).

### Putative aquatic-habitat associated spidroins in *A. aquatica* and *D. marina*

A previous study of spidroins from one semi-aquatic spider, *D. triton*, did not detect modifications associated with wet environments^37^. However, now with spidroin sequences from additional species, we observed a dramatically higher concentration of hydrophobic amino acid motifs in Sp sequences in the water-associated *A. aquatica*, *D. marina*, and *D. triton* (20–38% GV) compared to the terrestrial *B. longinqua* and *S. mimosarum* (2–4% GV). The prevalence of GV motifs in water-associated species could be an acquired mechanism for silk use in water.

GV motifs may be spider-specific. Indeed, we compared all the spidroins from our study to the silks of other arthropods that also live in aquatic habitats and found little sequence similarity. Silks from non-spider aquatic arthropods also have proteins (that are not spidroins) with repetitive motifs that have been argued as playing an important role in survival and reproduction^63^. For example, caddisfly larvae (Trichoptera) live in freshwater and spin protective cases and capture webs. The main structural protein in their silk is heavy chain fibroin, which has serine-rich motifs (SXSXSX) that interact with divalent ions^64^. This interaction is thought to be essential for underwater adhesion. We found no evidence for similar serine-rich motifs in the spidroins of aquatic spiders (Figs 4 and S1, S4).

Similar to caddisfly larvae, amphipods, and marine worms have molecular specializations to marine environments. For example, the adhesive threads of the tube-building corophioid amphipod have a high proportion of basic residues, especially arginine^65^; the proteinaceous glue in a polychaete marine worm has proteins enriched with XGGYGYGGK repeat motifs and phosphorylated serine^66^. We searched our sequences from aquatic and semi-aquatic spider species for features associated with marine adhesion but found no similarities to sequences from amphipods or marine worms. We did not find evidence of the convergent evolution of sequence elements used by other aquatic arthropods. Instead, we found that the spidroin sequences from water-associated spiders have high concentrations of the hydrophobic GV motif. Characterization of spidroins from additional species is needed to test whether there is a correlation between aquatic/non-aquatic habitats and presence/absence of the GV motif.

### Sex-Associated differences in spidroin gene expression

**Figure 5:**
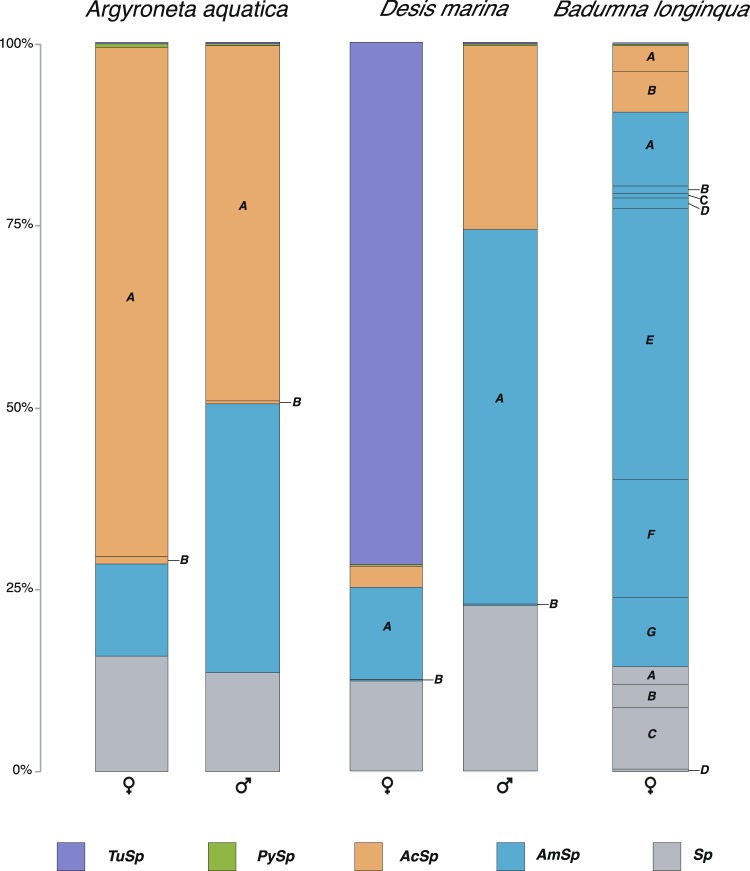
Relative expression levels of spidroin genes. Stacked bar graphs of gene expression levels in *Argyroneta aquatica* females (left) and males (right), *Desis marina* females (left) and males (right), and *Badumna longinqua* females. *TuSp* (purple), *PySp* (green), *AcSp* (orange), *AmSp* (blue), and *Sp* (grey) are shown. Letters indicate different variants for each spidroin type (e.g. *AcSp_vA* and *AcSp_vB* are indicated by the *A* and *B* in orange). Percentages show reads per kilobase of transcript per million mapped reads (RPKM) of average expression of male and female reads mapped to species-specific transcriptomes. Total RPKM of spidroins for *A. aquatica*: ♀ 2,883, ♂ 5,041; *D. marina*: ♀ 11,260, ♂ 8,155; and *B. longinqua* ♀ 43,284.

Relative levels of spidroin gene expression were quantified by mapping reads from each silk gland RNA-Seq library to its respective transcriptome assembly (Fig. 5). For all three species, there were silk gland libraries from females. For *A. aquatica* and *D. marina*, there were also silk gland libraries from males.

*AcSp* genes have the highest relative expression of all spidroins in *A. aquatica* females and males (Fig. 5). Specifically, one variant (*A. aqu*_*AcSp*_*N_vA*) accounted for 70% of total spidroin expression in females and 49% of males. The second highest expressed spidroins are the *AmSp* group. Although we found both male and female *A. aquatica* spiders to have *AmSp* genes as the second highest expressed spidroin type, ampullate spidroin expression is over five-fold higher in males (1,866 RPKM) than in conspecific females (364 RPKM). Observations of web construction by male and female *A. aquatica* spiders have documented that males use more dragline silk during web construction when compared to females^29^. This observation is consistent with our expression data, assuming that AmSp is a major constituent of dragline threads (Fig. 5).

While *AmSp* gene expression was the second highest in *A. aquatica*, *AmSp* gene expression was by far the highest in *B. longinqua* (76%; Fig. 5). The majority of this expression was driven by one *AmSp* variant, *B. lon_AmSp*_*N_vE* (37% of total spidroin expression). *B. longinqua* is a cribellate spider and prey-catching webs of other cribellate spiders have been observed to be composed of at least two different silk fibers, one fiber corresponding to ampullate silk and the other to cribellar silk^67^. This suggests that *B. lon_*AmSp_N_vE is the main protein being produced by *B. longinqua* spiders for dragline silk and their prey-catching webs.

Ampullate spidroin genes were also the most highly expressed spidroin type in *D. marina* males (Fig. 5). In *D. marina* females, however, tubuliform (egg case) spidroins had the highest expression relative to other spidroin genes. Tubuliform glands are limited to females and thus males are not expected to express tubuliform spidroins. After spiders become sexually mature, female investment shifts from feeding to the production of egg cases, which are mainly composed of tubuliform silk. Mature males, by contrast, shift to a roving lifestyle in search of receptive females. Expression profiles of *D. marina* males and females are consistent with sex-specific changes after sexual maturation with females having a higher expression of *TuSp* (egg sac) spidroin gene and males expressing spidroins associated with movement (*AmSp* and *AcSp*). This pattern is also consistent with expression patterns described for cob-web weaving spiders^2^.

### Composition of retreats and webs for aquatic environments

*Desis marina* spiders live in the intertidal zone, where they construct silken retreats in which they store a bubble of air to withstand submersion by seawater for long periods of time^19^ (Fig. 1A). Both male and female *D. marina* spiders make these silk retreats. Therefore, we would expect silk gene expression levels to correlate with silk use. We found *AmSp*, *Sp*, and *AcSp* genes combined to account for 28% and 99% of expression in females and males respectively (Fig. 5). We therefore propose that *D. marina* spiders use silks composed of these spidroins to build their retreats. This scenario is consistent with a previous report that showed *D. marina* silk retreats are made of two types of fibers: a main fiber with a diameter of 1.54 μm and a second fiber with a diameter of 0.38 μm^22^. Fiber diameter tends to correspond with spigot size; for example, major ampullate spigots are larger in size than aciniform spigots and major ampullate fibers are larger in diameter than aciniform fibers. It is likely that *D. marina* spiders use ampullate silk as the main fiber and aciniform as the secondary fiber in their silken repeats. It is also possible that the primary fiber is composed of the “Sp” spidroin. If so, this implies that the Sp-producing silk gland is connected to the modified spigot on the posterior lateral spinneret, which is similar in size to ampullate spigots^35^.

The underwater web of *A. aquatica* serves as an air reservoir and shelter where spiders spend most of their time (Fig. 1C). Scanning electron microscope (SEM) micrographs of *A. aquatica* diving bells revealed a mixture of threads as well as a substantial amount of coating (Supplementary Fig. S6). Previous SEM studies of *A. aquatica* webs have shown different types of threads of variable thickness, flat bundles 200–400 μm across, and a proteinaceous gel-like mass or hydrogel embedding all threads^68^.

An experiment to test the components of *A. aquatica* diving bell involved covering the opening of pyriform and major ampullate spigots on the anterior spinnerets with resin^36^. It was shown that treated spiders were able to construct a normal, functional diving bell but did not make the threads attaching the web to the surrounding vegetation. This suggests that the web is mainly produced by silk glands connected to spigots on the median and posterior spinnerets^36^ (i.e., minor ampullate and aciniform glands). Based on these observations, the different fiber morphologies observed in SEM micrographs (Supplementary Fig. S6), and the expression profile of *A. aquatica* with high expression of *AcSp*, *AmSp*, and *Sp* relative to all spidroins (Fig. 5), we posit that the bell is composed of aciniform, pyriform, ampullate, and Sp silk. This hypothesis is partially supported by proteomic analysis of the diving bell. We found AcSp, PySp, and Sp spidroins to be part of *A. aquatica* diving bell (Supplementary Table S2). This means that Sp is likely a component of minor ampullate silk and if so, is expressed in minor ampullate glands. It also suggests that *A. aqu*_AmSp_N is likely a component of attaching threads and produced in major ampullate glands connected to major ampullate spigots on the anterior spinnerets^36^. Ampullate silk has been observed to be used in web construction by *A. aquatica* spiders as the structural lines that attach the bell to aquatic plants^29^. However, we were unable to collect all the structural lines of the diving bell and so it is possible that our collected sample did not include ampullate fibers, explaining why AmSp was not recovered in proteomic analyses.

## Summary

We identified 47 spidroin transcripts in the assemblies from the focal species (Supplementary Table S1). These transcripts correspond to aciniform, cribellar, pyriform, tubuliform, and ampullate spidroins, plus a novel type of spidroin, the GV-rich Other Spidroin. Aciniform, pyriform, and tubuliform spidroins from *A. aquatica*, *B. longinqua*, and *D. marina* are fairly conserved in the repetitive and terminal regions across species and when compared to previously described spidroins (Figs 2 and 3, Supplementary Fig. S1), consistent with similar selective pressures shaping spidroins independent of whether they are used in terrestrial only, semi-aquatic, or aquatic environments.

*B. longinqua* CrSp supports a cribellate specific spidroin hypothesis. A spidroin recently identified to be associated with cribellar silk in the spider *T. perfuga* (CrSp^59^) was also discovered in the cribellate spider *B. longinqua*. CrSp repeat units from both species are highly conserved in sequence (Supplementary Fig. S3). Unlike *B. longinqua*, *A. aquatica* and *D. marina* do not have cribellums, do not produce cribellar silk, and CrSp was not found in their transcriptome assemblies. This is further evidence associating *B. longinqua* CrSp specifically with cribellar silk production.

Although our spidroins show diversity in repeat composition (Supplementary Figs S1 and S4), sequence comparison of aciniform, pyriform, tubuliform, and ampullate spidroins do not show unique modifications for semi-aquatic environments. However, we identified a highly hydrophobic amino acid motif, GV, only in the spidroins of spider species associated with wet environments (Fig. 4). Based on this association, we hypothesize that the amino acid motif (GV) is involved in the efficiency of underwater webs and retreats to withstand submersion.

Polymers with high content of non-polar amino acids arranged on repeating motifs are especially desirable for tissue engineering applications. For example, synthetic polypeptides of the elastin repeating motifs VGVPG or VGGVG^69^ have been synthesized and chemically cross-linked to produce hydrogels with a range of mechanical properties and permeability^70–72^. *Argyroneta aquatica* spiders make a hydrogel in part composed of AcSp and Sp, and thus the cross-linking of GV rich repeating unit found in Sp could contribute to the diving bell’s permeability, allowing for oxygen diffusion.

Within Dictynoidea, there are a large number of spiders that occur along the seashore or near freshwater. These are habitats that only a few other spider groups utilize to a similar degree (e.g. Lycosidae and Pisauridae^15,73^). Aquatic Dictynoidea spiders have characteristics such as the extension of the tracheae to the cephalothorax^15^ and hydrophobic hairs that allow for plastron formation and immersion resistance^74^. We show that a spidroin type with an unusual concentration of hydrophobic residues may also contribute to the ability of aquatic and semi-aquatic spiders to thrive in aquatic and semi-aquatic environments.

## Material and Methods

### Construction and sequencing of RNA-Seq libraries

Adult female *B. longinqua* were collected in Vista (San Diego County), California, USA. Silk glands were extracted from each individual, flash frozen, and stored at −80 °C. Adult female and male *D. marina* were collected in Kauri Point Reserve, New Zealand. Adult female and male *A. aquatica* were collected in Neerpelt, Belgium. The cephalothoraxes (without venom glands) and silk glands were dissected from each individual *D. marina* and *A. aquatica* and the tissues were immediately submerged in RNALater (Sigma-Aldrich, Milwaukee, WI, USA). Each cephalothorax had the venom glands removed to obtain a single type of non-silk gland control tissue. Silk glands for each individual were separated from the surrounding fatty and reproductive tissue taking care to avoid any rupturing of the silk glands. Moreover, silk glands and their ducts were kept intact by keeping them attached to their corresponding spigot. Individual RNA extractions were done for all tissues from each spider following the methods of Starrett *et al*.^7^. Quantification of each RNA extractions was done using a Qubit Fluorometer (Thermo Fisher Scientific, Wilmington, MA, USA) and RNA integrity was assessed with a Bioanalyzer (Agilent 21000, Agilent Technologies, Santa Clara CA, USA). Twelve RNA-Seq libraries were constructed with the Ovation Universal RNA-Seq System (NuGen, San Carlos, CA, USA). *Argyroneta aquatica* and *D. marina* libraries were made from the following tissues: two sets of female silk glands, two sets of male silk glands, and two cephalothoraxes, for a total of six libraries per species. Libraries made from *B. longinqua* included two sets of female silk gland tissues. Each library was depleted of species-specific ribosomal rRNA using InDA-C probes. Bidirectional sequencing of the libraries (2 × 150 bp, mid-output) was done on a NextSeq 500 System (Illumina) at the University of California, Riverside Genomics Core Facility.

### Transcriptome assembly and estimates of expression level

Low-quality reads and adaptors were removed from raw sequencing reads using Trimmomatic^75^. FastQC was used to evaluate the quality of the resulting filtered reads (Babraham Bioinformatics FastQC Package**)**. All reads from the same species were combined for *de novo* assembly of species-specific transcriptomes using Trinity v2.1.1 with default parameters^76^. For assembly statistics see Supplementary Table S3. Transcriptome quality was approximated with N50 and completeness evaluated by comparison to the arthropod v9 set of Universal Single-Copy Ortholog (BUSCO v 3.0^77^). We identified 91% and 97.5% of the *Ixodes* BUSCOs as complete in the *A. aquatica* and *D. marina* assemblies, respectively, which were built from three tissue types (cephalothorax, female silk glands, and male silk glands). The *B. longinqua* assembly was less complete (66%), as expected because it was built from only one tissue type (female silk glands). Putative bacterial sequences, chimeras, and sequencing errors were identified and removed from each assembly based on BLASTX results using the methodology described in Clarke *et al*.^40^.

Filtered reads from each species were mapped to their corresponding species-specific assemblies with TopHat2 v2.1.1 using default parameters^78^. For each transcript, we calculated Reads Per Kilobase per Million mapped reads (RPKM). A minimum of ten reads mapped and more than one RPKM in at least two assemblies was used as a cut-off for inclusion in the gene expression analyses. Similar expression patterns were observed whether using transcripts containing the N- or C-terminal encoding region. Counts from the two regions were not combined because we could not definitively match each N-terminal region transcript with a counterpart C-terminal transcript. Instead, counts to transcripts containing the N-terminal region were used. We found biological replicates from each species to have strong positive Pearson’s correlations between 0.88 to 0.99. Principal component analyses show tissues used in the study clustering based on identity and tissue type (data not shown).

### Spidroin annotation and phylogenetic analyses

A silk protein database composed of spidroin genes, egg case silk proteins (ECP-1 and ECP-2), spider coating peptides (SCP-1 and SCP-2), and aggregate silk factors (AgSF1 and AgSF2) was constructed from downloaded UniProtKB/Swiss-Prot and NCBI nr protein databases in March 2017 (see Supplementary Table S4). This database was used to identify silk genes via BLASTX searches with e-value < 1 e-5 in Geneious v8.1.8. Only spidroin transcripts were found, there were no hits to spider coating peptides, egg case silk proteins, or aggregate silk factors. Sequences with BLAST hits to spidroins were inspected for the presence of known regions of spidroin genes, specifically for the presence of conserved terminal domains (N and C) and spidroin-specific repetitive regions (Supplementary Fig. S1A). Transcripts were considered to represent the same locus by having >90% nucleotide identity and are represented by the longest transcript.

Terminal encoding regions (N and C) from spidroin contigs identified from each species were translated and pooled with other araneomorph (true spider) published spidroin sequences. Araneomorph spidroins were chosen to represent a wide diversity of the spidroin family (Supplementary Table S5). Each set of terminal regions were aligned separately with MUSCLE implemented in Geneious and the resulting alignment was polished by eye. Amino acid model test and Maximum likelihood analyses done using RAxMLv8.2.8^79^. JTT and WAG amino acid model tests were used for N- and C-terminal alignments respectively. Resulting trees with 10,000 BT replicates were visualized using FigTree v1.4.3 (http://tree.bio.ed.ac.uk/software/figtree/).

### Proteomic analysis and SEM of *Argyroneta aquatica* diving bells

*A. aquatica* spiders were individually housed in aquariums. Fresh diving bells were harvested for proteomic analyses and scanning electron microscopy (SEM). Webs were taken out of the water and immediately processed. Extractions of proteins and processing of peptides were done according to Chaw *et al*.^80^. In short, individual webs were cut into smaller pieces using a sterile micro-scissors and submerged in protein extraction buffer with Halt protease inhibitor cocktail (Thermo Fisher Scientific, Waltham, MA, USA). Samples were homogenized with a sterile pestle and incubated overnight at room temperature. Samples were separated on an SDS-PAGE gel and proteins were digested using the in-gel tryptic digest protocol for in-gel digestion from Arizona Proteomics Consortium (http://proteomics.arizona.edu/protocols). Proteins were extracted from the gel and later purified with Ziptips C18 pipette tips (Millipore, Billerica, CA, USA).

LC-MS/MS analysis was done at the University of Arizona’s Arizona Proteomics Consortium on an LTQ Orbitrap Velos mass spectrometer (Thermo Fisher Scientific) suited with a nanomate ESI source (Advion, Ithaca, NY, USA). Using the Thermo Proteome Discoverer 1.3 (Thermo Fisher Scientific), all resulting spectra were matched against three databases: Chelicerata proteins downloaded from NCBI (on October 17, 2013), a common contaminant proteins database, and non-redundant longest open reading frame translation of our *de novo A. aquatica* transcriptome. The proteome software Scaffold v4.7.3 (Proteome Software Inc., Portland, OR, USA) was used to visualize protein and peptide identification results. Resulting proteins with at least one peptide that was identified with 95% peptide confidence and 95% protein confidence were accepted.

Freshly made *A. aquatica* webs were cut into 8 × 5 × 3 mm sections using sterile micro-scissors. Each section was anchored to aluminum pin stubs using carbon tape, and platinum-palladium was used for coating in preparation for Scanning Electron Microscope. Micrographs were obtained using a Mira3 SEM system (Tescan, Czech Republic) with a 5–10 kV accelerating voltage at the University of California, Riverside Central Facility for Advanced Microscopy and Microanalysis.

## Supplementary information

Supplementary Information

## Data Availability

All raw sequencing reads are deposited in the NCBI Short Read Archive and transcriptome is deposited at the DDBJ/EMBL/GenBank Transcriptome Shotgun Assembly database. All data is avaibale under the BioProject numbers PRJNA510262, PRJNA510260, and PRJNA510264 for *Argyroneta aquatica*, *Badumna longinqua*, and *Desis marina* respectively.
