# Supplementary material for: Spidroins and Silk Fibers of Aquatic Spiders
Source: Sci Rep. 2019 Sep 20;9:13656. doi: 10.1038/s41598-019-49587-y (PMC6754431; doi:10.1038/s41598-019-49587-y)
Supplement: Supplementary file 1 — Supplementary Information [file 41598_2019_49587_MOESM1_ESM.pdf]

Spidroins and Silk Fibers of Aquatic Spiders

Sandra M. Correa-Garhwal, Thomas H. Clarke III, Marc Janssen, Luc Crevecoeur, Bryce N. McQuillan, Angela H. Simpson, Cor J. Vink, Cheryl Y. Hayashi

Supplementary Text

Supplementary Results and Discussion

Aciniform spidroins have mostly been described from orb-web and cob-web weaving spiders, and are considered the main components of aciniform silk[1–4]. Aciniform silk is used for web construction, web decoration, and wrapping of prey. Similar to aciniform spidroins from other species[3–6], the AcSp protein sequences from our three focal species have long repeats (ranging from 173-206 aa; Supplementary Fig. S1). *Badumna longinqua* and *Argyroneta aquatica* each have at least two AcSp variants, which we named variants A and B (AcSp_vA and AcSp_vB). The *B. longinqua* AcSp variants have different repeat units, sharing only 41% sequence similarity at the amino acid level, suggesting that there are at least two AcSp loci in the *B. longinqua* genome. For the *A. aquatica* AcSp variants, only one transcript contained a complete repeat unit (*A. aqu*_AcSp_N_vB; Supplementary Fig. S1B). However, comparison of the available repetitive regions of *A. aquatica* AcSp variants shows few similarities with only 25% sequence identity (over 80 aa), also suggesting that *A. aquatica* has two AcSp loci. This finding is consistent with previously published *A. aquatica* silk sequences, where at least two AcSp transcripts were reported[7].

Female spiders protect their eggs by wrapping them in silk. The silken egg cases are mainly composed of tubuliform silk[8–10]. Each of our focal species contained transcripts that were long enough to have more than one complete repeat unit of TuSp (Supplementary Figure S1C). The TuSp repeat units were very similar across species, with 190 aa in *A. aquatica,* 194 aa in *B. longinqua*, and 196 aa in *Desis marina*. Repeat units from all three species are comparable in length to the 180–184 aa TuSp1 repeat units from the cob-web weaving *Latrodectus hesperus*[11] and the orb-web weaving spiders *Argiope bruennichi*[12] and *Argiope argentata*[13]. Not only are the TuSp repeat units conserved in length across species, they have conserved amino acid sequence motifs. The amino acid sequence motifs poly-serine, poly-alanine, and glycine-X (where X could be A, I, L, Q, S, T, V, or Y), are common in our focal species. Unlike the single, long poly-alanine motifs found in each MaSp1 repeat unit[14,15], poly-alanine motifs in TuSp are no more than four residues long and are dispersed across the sequence (Supplementary Figure S1C). The *A. aquatica* TuSp repeat also shares a five threonine (boxed, Supplementary Figure S1C) stretch with the TuSp repeats of the orb-web weaving spiders *Argiope aurantia*, *Nephila clavipes*, and *Araneus gemmoides*[16], the cob-web weaving spider *L. hesperus*[11], and the velvet spider *Stegodyphus mimosarum*.

References

1. Hayashi, C. Y., Blackledge, T. A. & Lewis, R. V. Molecular and Mechanical Characterization of Aciniform Silk: Uniformity of Iterated Sequence Modules in a Novel Member of the Spider Silk Fibroin Gene Family. *Mol. Biol. Evol.* **21**, 1950–1959 (2004).
2. Vasanthavada, K. *et al.* Aciniform spidroin, a constituent of egg case sacs and wrapping silk fibers from the black widow spider *Latrodectus hesperus*. *J. Biol. Chem.* **282**, 35088–35097 (2007).
3. Ayoub, N. A., Garb, J. E., Kuelbs, A. & Hayashi, C. Y. Ancient properties of spider silks revealed by the complete gene sequence of the prey-wrapping silk protein (AcSp1). *Mol. Biol. Evol.* **30**, 589–601 (2013).
4. Chaw, R. C. *et al.* Intragenic homogenization and multiple copies of prey-wrapping silk genes in *Argiope* garden spiders. *BMC Evol. Biol.* **14**, 31 (2014).
5. Correa-Garhwal, S. M. *et al.* Silk genes and silk gene expression in the spider *Tengella perfuga* (Zoropsidae), including a potential cribellar spidroin (CrSp). *PLOS ONE* **13**, e0203563 (2018).
6. Correa-Garhwal, S. M. *et al.* Semi-aquatic spider silks: transcripts, proteins, and silk fibres of the fishing spider, *Dolomedes triton* (Pisauridae). *Insect Mol. Biol.* 1–17 doi:10.1111/imb.12527
7. Strickland, M., Tudorica, V., Řezáč, M., Thomas, N. R. & Goodacre, S. L. Conservation of a pH-sensitive structure in the C-terminal region of spider silk extends across the entire silk gene family. *Heredity* 1 (2018). doi:10.1038/s41437-018-0050-9
8. Hu, X. *et al.* Spider egg case core fibers: trimeric complexes assembled from TuSp1, ECP-1, and ECP-2. *Biochemistry* **45**, 3506–3516 (2006).
9. Hu, X. *et al.* Egg Case Protein-1 a new class of silk proteins with fibroin-like properties from the spider *Latrodectus Hesperus*. *J. Biol. Chem.* **280**, 21220–21230 (2005).
10. Casem, M. L., Collin, M. A., Ayoub, N. A. & Hayashi, C. Y. Silk gene transcripts in the developing tubuliform glands of the Western black widow, *Latrodectus hesperus*. *J. Arachnol.* **38**, 99–103 (2010).
11. Hu, X. *et al.* Araneoid egg case silk: a fibroin with novel ensemble repeat units from the black widow spider, *Latrodectus hesperus*. *Biochemistry* **44**, 10020–10027 (2005).
12. Zhao, A.-C. *et al.* Novel molecular and mechanical properties of egg case silk from wasp spider, *Argiope bruennichi*. *Biochemistry* **45**, 3348–3356 (2006).
13. Chaw, R. C., Collin, M., Wimmer, M., Helmrick, K.-L. & Hayashi, C. Y. Egg case silk gene sequences from *Argiope* spiders: evidence for multiple loci and a loss of function between paralogs. *G3 GenesGenomesGenetics* **8**, 231–238 (2017).
14. Ayoub, N. A., Garb, J. E., Tinghitella, R. M., Collin, M. A. & Hayashi, C. Y. Blueprint for a high-performance biomaterial: full-length spider dragline silk genes. *PloS One* **2**, e514 (2007).
15. Hinman, M. B. & Lewis, R. V. Isolation of a clone encoding a second dragline silk fibroin. *Nephila clavipes* dragline silk is a two-protein fiber. *J. Biol. Chem.* **267**, 19320–19324 (1992).
16. Tian, M. & Lewis, R. V. Molecular characterization and evolutionary study of spider tubuliform (eggcase) silk protein. *Biochemistry* **44**, 8006–8012 (2005).

Supplementary Figures

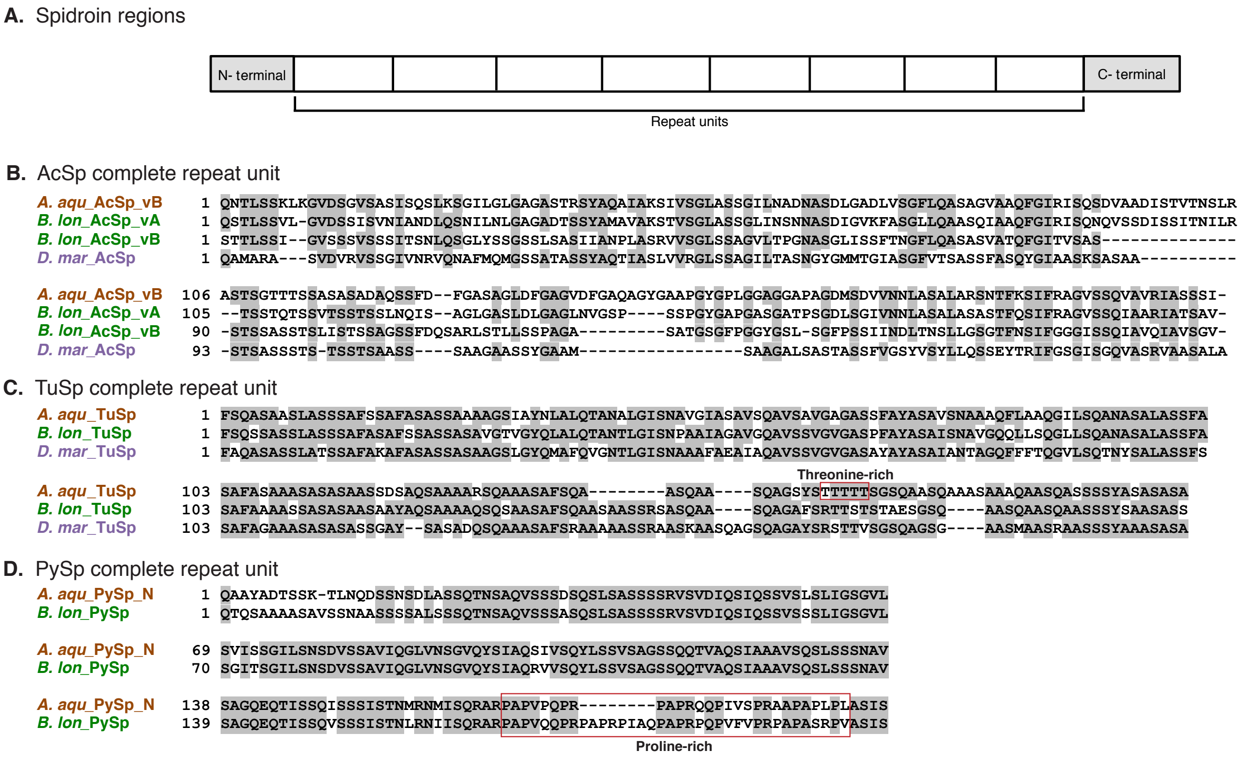

Figure S1. Exemplar repeat units for aciniform, tubuliform, and pyriform spidroins. Schematic organization of a spidroin primary structure showing the tandemly arrayed repeat units (**A**). Multiple alignments comparing single repeat units of aciniform spidroins (AcSp; **B**), tubuliform spidroins (TuSp; **C**), and pyriform spidroins (PySp; **D**) of *Argyroneta aquatica*, *Badumna longinqua*, and *Desis marina*. Sequence names highlighted as in Figure 2 and abbreviated as in Supplementary Table S1. Amino acids conserved > 50% across all sequences are highlighted in grey. Gaps inserted into the alignment are indicated by dashes and missing sequence by periods. Amino acid positions for each sequence are numbered on the left. Proline and threonine-rich regions shown in red boxes.

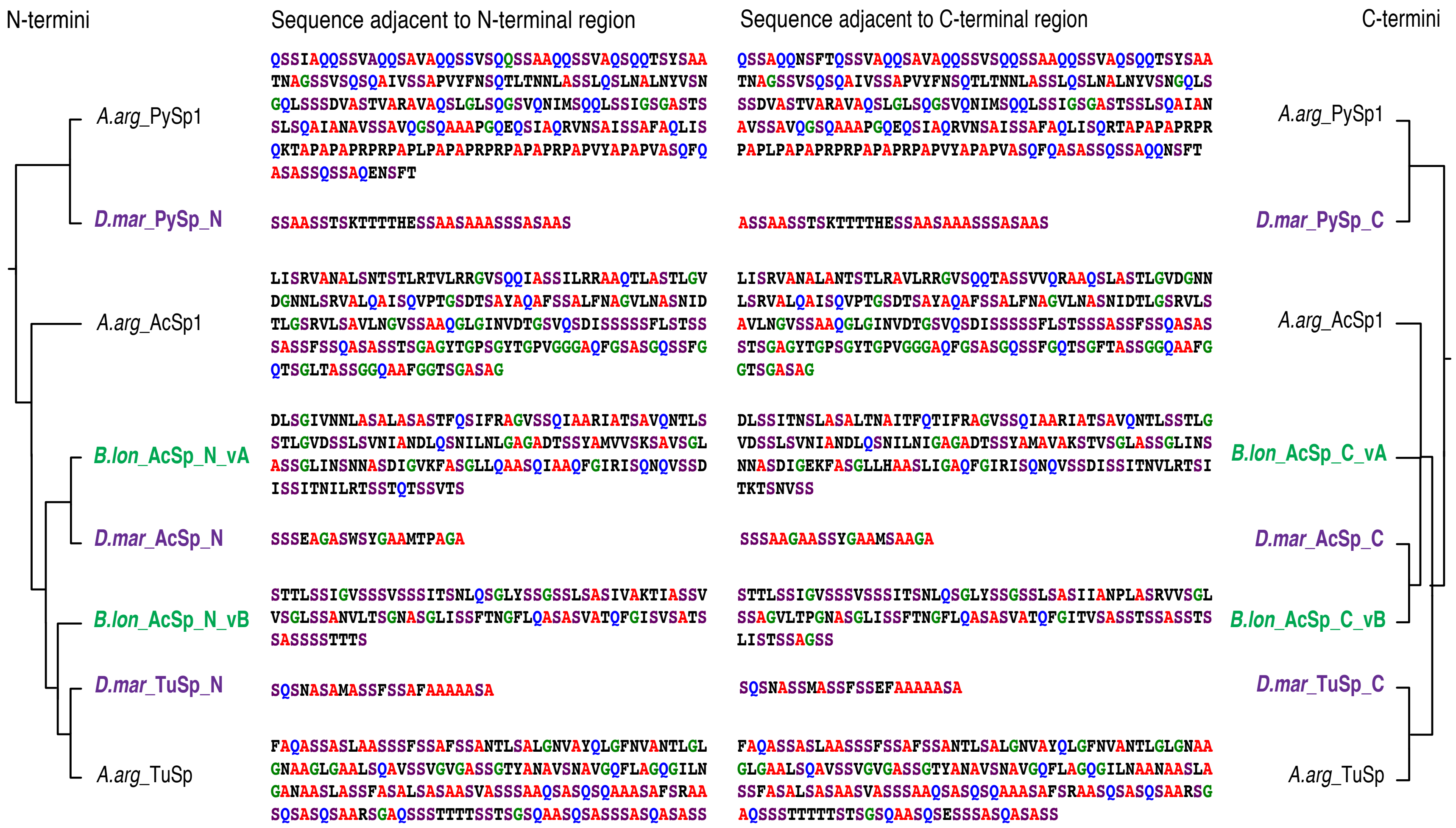

Figure S2. Comparison of repeats from transcript sequences towards the beginning or end of the same spidroin. Sequence names highlighted as in Figure 2 and abbreviated as in Supplementary Tables S1 and S5. Abundant amino acids highlighted: alanine (red), serine (purple), glycine (green), and glutamine (blue). Cladograms reflect the relationships in Figures 2-3 for N- and C-terminal regions, respectively

```
B.lon_CrSp   1 AVGSHLYETLLSNPRFVSSFGLEFSLAKARVFLSALASRMHSFPQFSSLRVQDLVKRYLDALETITLGSSVSLYAQTIS
S.mim_CrSp   1 AFGSHLYGTLLVNPRFSTLFGSEFSLEKVRPFLFALASHIHSFSQFSSISANDLFERYIEVVNALPLGSSVQAYALALS
T.per_CrSp   1 AFGSHLYGTLLVNPRFVTVFGSDFSLERSRLFLSVLSSRIHSFPQFSSIPVQYLLNRYTDVVASIPFGSSEQIYARRIA

B.lon_CrSp  80 QVTASFLKESNLLSWQLISDKYEAIDEATSEAVESIIETTPLTEKSLSTGLPSVEDSAATTAATAVFSPSVLHVLSTAE
S.mim_CrSp  80 QATAELLYENNLLSWDALAKEDAEAAGAG-EAQATVSSTLVS---------SSTVESAAAETAASAILSPSVLSILSSSE
T.per_CrSp  80 QETASVLYKNNLLSWQILASEDAAVDKAA-EDAGAVLSQEASLSDQSISLSSSTEDVAASMAASAVLSPSVLETLATAE
```

Figure S3. Cribellar spidroin (CrSp) repeat unit. Identified CrSp motif of *Badumna longinqua* (green) aligned to *Tengella perfuga* (*T.per*_CrSp), and *Stegodyphus mimosarum* (*S.mim*_Sp1; GenBank: KFM60634.1). Names abbreviated as in Tables S1 and S4. Amino acids conserved 100% across all sequences are shaded in grey. Abundant amino acids in silks are highlighted: alanine (red), serine (purple), glycine (green), leucine (orange), and glutamine (blue). Gaps inserted into the alignment are indicated by dashes. Amino acid positions for each sequence are numbered on the left.

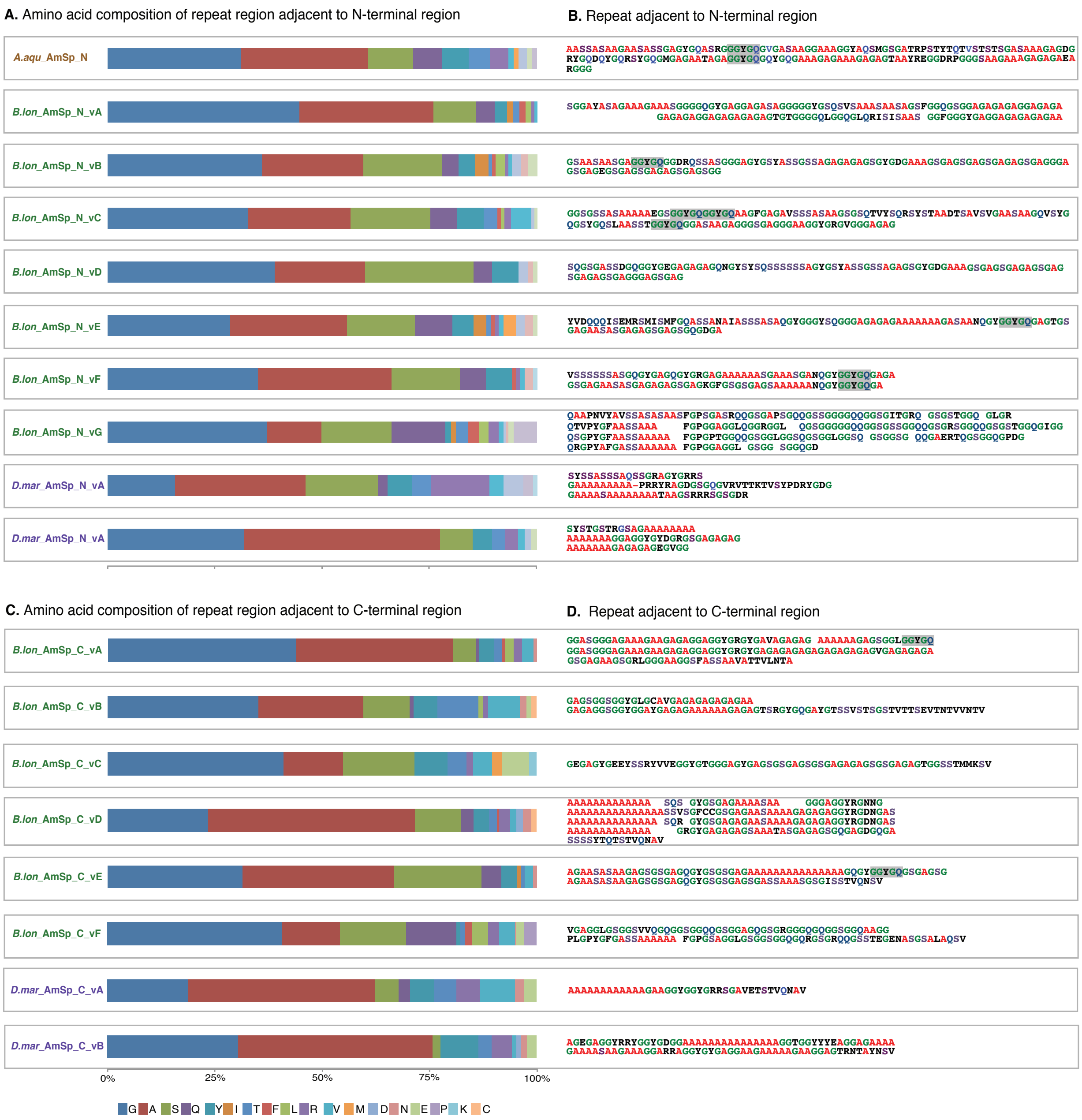

Figure S4. Repetitive regions of Ampullate Spidroin (AmSp) sequences. Amino acid composition of AmSp repetitive regions (**A** and **C**) adjacent to the N-terminal (**B**) or C-terminal (**D**) regions of *Argyroneta aquatica, Badumna longinqua*, and *Desis marina*. Sequence names abbreviated as in Supplementary Table S1 and highlighted as in Figure 2. Amino acids abundant in AmSp sequences are highlighted as in Figure S2. The amino acid motif GGYGQ, which is found in several sequences, is highlighted by grey shading.

```
B. lon_Sp_N_vB    1 NALINDESFNSAIQTGISSTSISALASAIAGSVASAPEFSAVGYSALVKAYLQAISDEKEGATVSDYARGIAAATTSVLDNAGIFAAGIDLEHITAAVGAIASGIHSTSGIQAVSDSHVTPGDASNV
S. mim_Sp2b       1 NALINDSGFQSAFSGTVSTNIINAFATAIAGSVASASEFSSVGYSSLVKAYLQALSSEKDGATIEDYARAIATATSNVLEQNGIFSEGVASGHITAAVNAITSGISSTH----ITESAVTTTSVGSQ

B. lon_Sp_N_vB  128 LTAAVTTATVDGSSPVSHIPAYTALTGTPAISISFARQIYAALLADPQFGLSFQQPISLERIRLHLTAIATSITSIPQYSLIDTNDLLNSYLDSIIGIPPGSSTFVYAQAIARVTAAVLFKHDLLTW
S. mim_Sp2b     124 TTVSSDAGTSSG---VGHIPAYTAPAGTPAVAINFARQIYISLLADPSFSSVFQAPISLDRVKIYLAALAKFIVAIPRYSIVSADELVNGYLGTIIGIPPGSASSIYAQAIARITADTFYKNGLLSL

B. lon_Sp_N_vB  255 ESVNTGLPEVQNAIQSALVSDTTSSENDVESSIDTRATSKDLTLVTTGLKGEEAGTGDLYQTTLTPETAPSQIKEPSVLEIRDQYVAPEGTPAVSVAFAKKIYAALLSDNRFITAFESPLSIPRARI
S. mim_Sp2b     248 DTVNAESGIIQNAVQNAL-----------------RSAALESSAITSGTEQTSADQKPISEHTIDD----------------HPYIAPEGTPAVSVAFAKRIYLALATDKRFVAAFTEPLSLTRARI

B. lon_Sp_N_vB  382 YLSAIAENLCALPRFNTINDEELVEGYVEAISSVPSEADVSIYAQEIATDTALVLFENNLLTWQAVTAGSSALSTAITDALSTAAERDSSIISSSPSIQIPSSDDDINITTGTEQISERDGTLRSVY
S. mim_Sp2b     342 YLSALAKSLCALPRYNTISDEELVEGYIEAISAVQTAPNPELYAQEIATVTALIFYENNLLTWQALTAGSAALQGAIQSALNAAAEEDTLAFSAT-----VAQTSTTQISSESQTSAVSDSTTLTAY

B. lon_Sp_N_vB  509 SPPSGTTPVARTFGKQIYGYLTENARFSSVFGKEFSLQNARLFLTALATSISSLPPFSSVTVSELVGKYIQAISAVPLGSDFYAYAQVIAEATSEILSSRKLLTLQVVSALSSSIQAAVGSALES
S. mim_Sp2b     464 NPPVGSTPVARNFGRLIYNSLLADEKFSSVFGIGNSFVNIRLFLTTLATSICSFPQFSSVTVSELVGKYIQAITPIPQGSDIHQYAQAIAQATAEIMSSRKLLSLQGVTALSSSLESAISSALES
```

Figure S5. Pairwise alignment of *Badumna longinqua* Sp_N_vB (green) and *Stegodyphus mimosarum* (Sp2b, GenBank: KK117516.1) repetitive region. Names abbreviated as in Tables S1 and S5. Amino acids conserved 100% across all sequences are shaded in gray. Gaps inserted into the alignment are indicated by dashes. Amino acid positions for each sequence are numbered on the left.

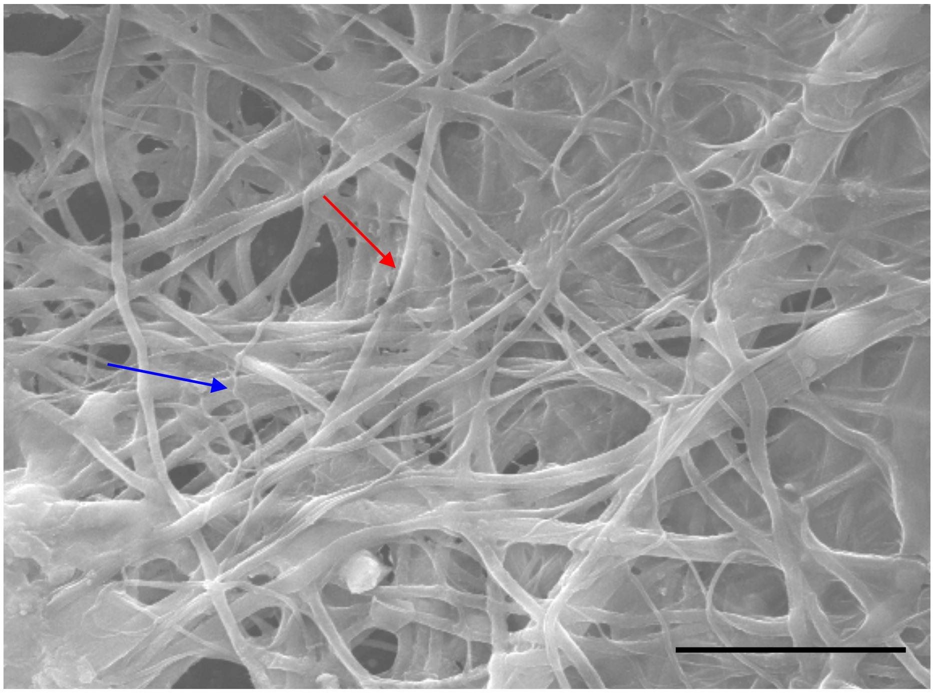

Figure S6. Scanning electron micrograph of *Argyroneta aquatica* diving bell. Large diameter fibers are indicated by a red arrow and smaller diameter fibers by a blue arrow. Scale bar 5 μm.

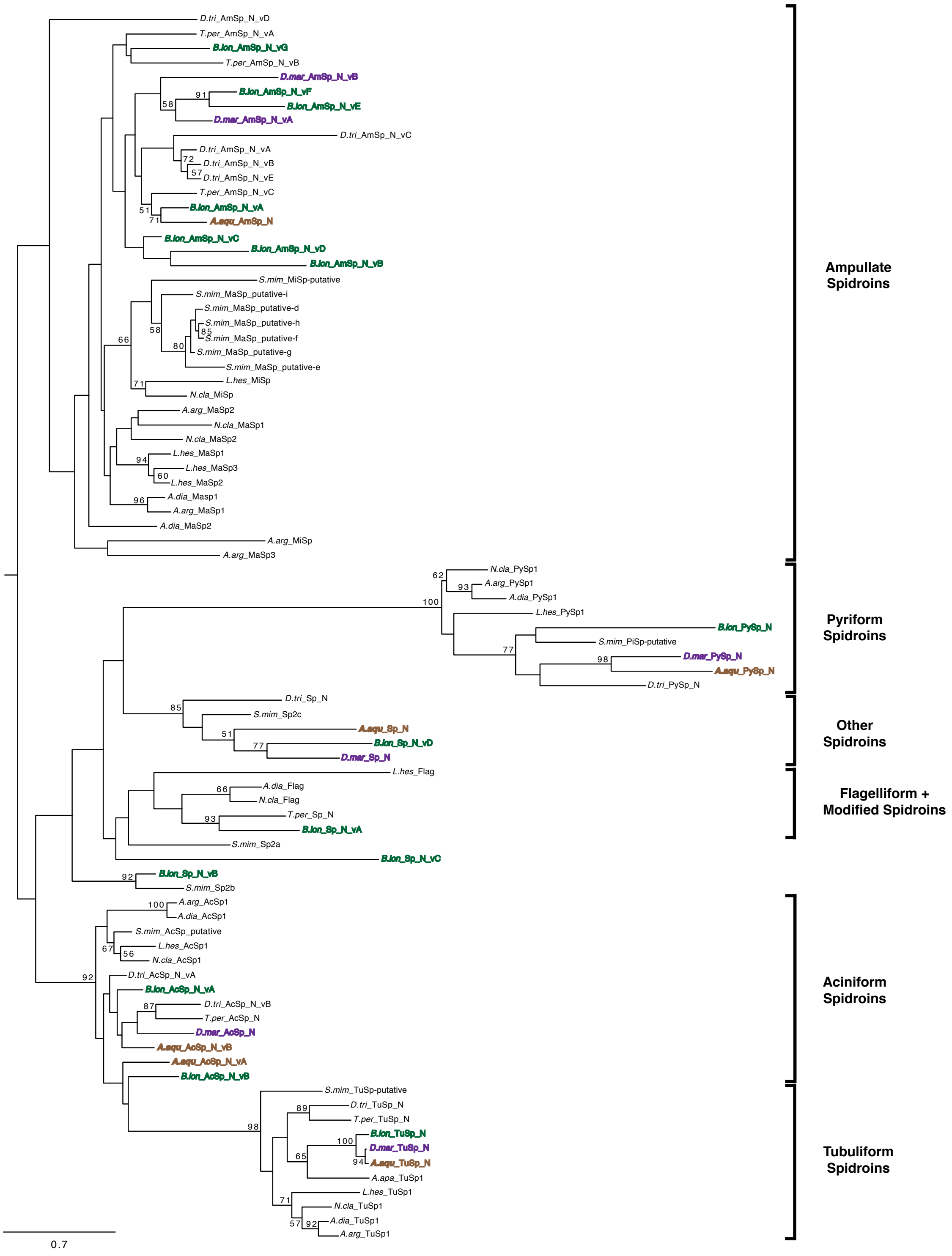

Figure S7. Complete maximum likelihood tree of spidroin N-terminal regions. *Argyroneta aquatica*, *Badumna longinqua*, and *Desis marina* spidroin paralogs highlighted in brown, green, and purple respectively. Tree rooted with the California trapdoor spider *Bothriocyrtum californicum* fibroin 1 (not shown). Names abbreviated as in Tables S1 and S5. Bootstrap percentages >50% are shown. Scale bar represents substitutions per site.

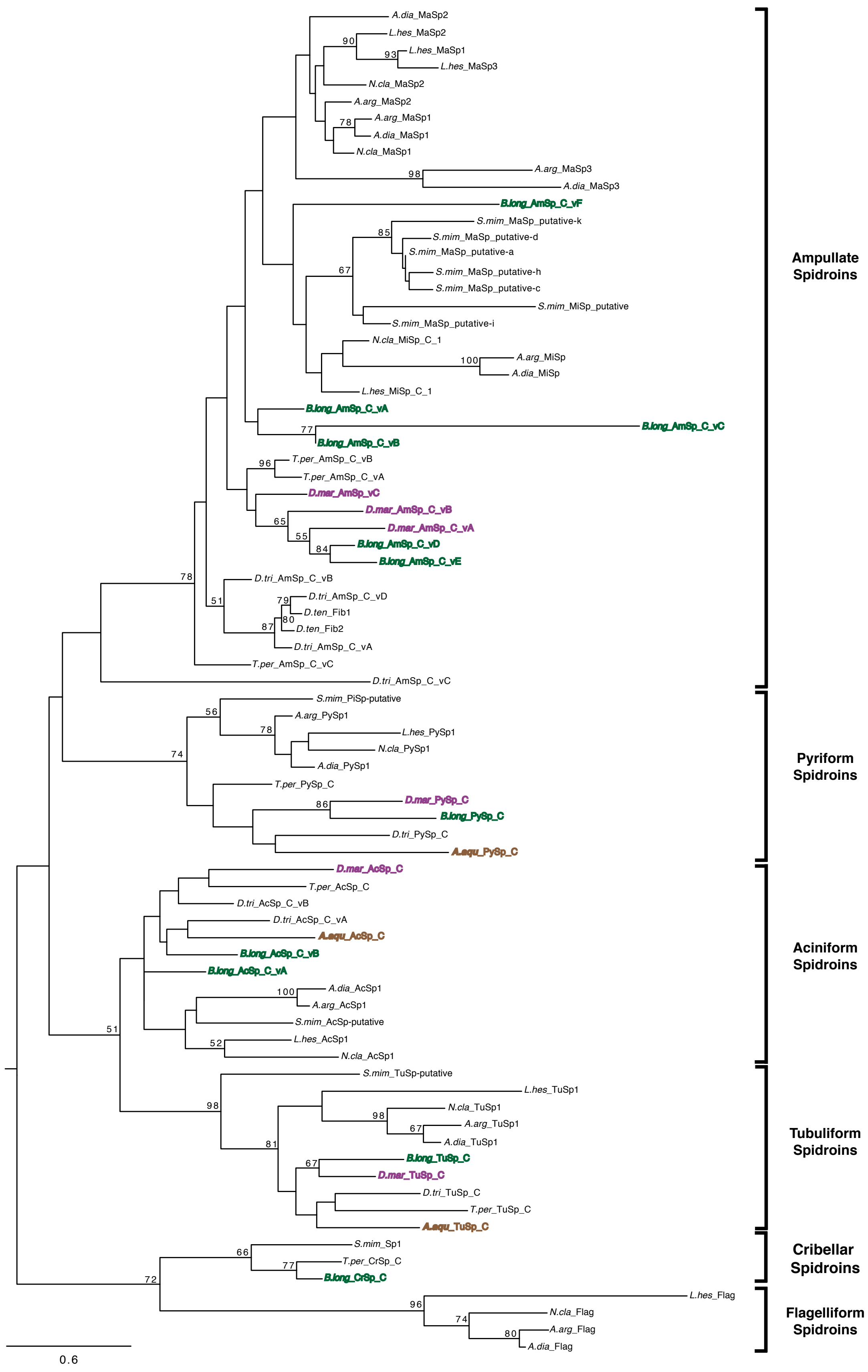
A.dia_MaSp2
L.hes_MaSp2
L.hes_MaSp1
L.hes_MaSp3
N.cla_MaSp2
A.arg_MaSp2
A.arg_MaSp1
A.dia_MaSp1
N.cla_MaSp1
A.arg_MaSp3
A.dia_MaSp3
B.long_AmSp_C_vF
S.mim_MaSp_putative-k
S.mim_MaSp_putative-d
S.mim_MaSp_putative-a
S.mim_MaSp_putative-h
S.mim_MaSp_putative-c
S.mim_MiSp_putative
S.mim_MaSp_putative-i
N.cla_MiSp_C_1
A.arg_MiSp
A.dia_MiSp
L.hes_MiSp_C_1
B.long_AmSp_C_vA
B.long_AmSp_C_vC
B.long_AmSp_C_vB
T.per_AmSp_C_vB
T.per_AmSp_C_vA
D.mar_AmSp_vC
D.mar_AmSp_C_vB
D.mar_AmSp_C_vA
B.long_AmSp_C_vD
B.long_AmSp_C_vE
D.tri_AmSp_C_vB
D.tri_AmSp_C_vD
D.ten_Fib1
D.ten_Fib2
D.tri_AmSp_C_vA
T.per_AmSp_C_vC
D.tri_AmSp_C_vC
S.mim_PiSp-putative
A.arg_PySp1
L.hes_PySp1
N.cla_PySp1
A.dia_PySp1
T.per_PySp_C
D.mar_PySp_C
B.long_PySp_C
D.tri_PySp_C
A.aqu_PySp_C
D.mar_AcSp_C
T.per_AcSp_C
D.tri_AcSp_C_vB
D.tri_AcSp_C_vA
A.aqu_AcSp_C
B.long_AcSp_C_vB
B.long_AcSp_C_vA
A.dia_AcSp1
A.arg_AcSp1
S.mim_AcSp-putative
L.hes_AcSp1
N.cla_AcSp1
S.mim_TuSp-putative
L.hes_TuSp1
N.cla_TuSp1
A.arg_TuSp1
A.dia_TuSp1
B.long_TuSp_C
D.mar_TuSp_C
D.tri_TuSp_C
T.per_TuSp_C
A.aqu_TuSp_C
S.mim_Sp1
T.per_CrSp_C
B.long_CrSp_C
L.hes_Flag
N.cla_Flag
A.arg_Flag
A.dia_Flag
90
93
78
98
85
67
100
77
96
65
55
84
78
51
79
80
87
56
78
74
86
100
52
51
98
98
67
81
67
66
77
72
96
74
80
0.6
Ampullate Spidroins
Pyriform Spidroins
Aciniform Spidroins
Tubuliform Spidroins
Cribellar Spidroins
Flagelliform Spidroins

Figure S8. Complete maximum likelihood tree of spidroin C-terminal regions. *Argyroneta aquatica*, *Badumna longinqua*, and *Desis marina* spidroin paralogs highlighted in brown, green, and purple respectively. Tree rooted with the California trapdoor spider *Bothriocyrtum californicum* fibroin 1 (not shown). Names abbreviated as in Tables S1 and S5. Bootstrap percentages >50% are shown. Scale bar represents substitutions per site.

Supplementary Tables

Table S1. Spidroins from this study for *Argyroneta aquatica, Badumna longinqua,* and *Desis marina*.

Spidroin Name[a]	Top BLASTx hit Accession	Top BLAST hit Description	E-value
***A. aqu*_AcSp_C**	gi|587655300|gb|AHK09813.1|	Aciniform spidroin 1 [*Argiope argentata*]	5E-04
***A. aqu* _PySp_C**	gi|257124471|gb|ACV41934.1|	Pyriform spidroin [*Latrodectus hesperus*]	9E-05
***A. aqu* _TuSp_C**	tr|A6YP77|A6YP77_9ARAC|	Fibroin 1 [*Hypochilus thorelli*]	1E-10
***A. aqu*_AcSp_N_vA**	gi|587655228|gb|AHK09777.1|	Aciniform spidroin 1 N-terminal region variant 2, partial [*Argiope trifasciata*]	1E-45
***A. aqu*_AcSp_N_vB**	gi|422900780|gb|AFX83566.1|	Aciniform spidroin 1, partial [*Latrodectus geometricus*]	1E-49
***A. aqu*_AmSp_N**	gi|193506893|gb|ACF19412.1|	Tubuliform spidroin 1, partial [*Agelenopsis aperta*]	9E-25
***A. aqu*_PySp_N**	gi|675382271|KFM75168.1|	Hypothetical protein X975_11824, partial [*Stegodyphus mimosarum*]	2E-18
***A. aqu*_Sp_N**	gi|675382271|KFM70693.1|	Hypothetical protein X975_03452, partial [*Stegodyphus mimosarum*]	9E-16
***A. aqu*_TuSp_N**	gi|303307781|gb|ADM14330.1|	Egg case silk protein 1 [Argiope bruennichi]	5E-32
***B. lon*_AcSp_C_vA**	gi|422900764|gb|AFX83559.1|	Aciniform spidroin 1, partial [*Latrodectus hesperus*]	6E-31
***B. lon*_AcSp_C_vB**	gi|422900780|gb|AFX83566.1|	Aciniform spidroin 1, partial [*Latrodectus geometricus*]	2E-20
***B. lon*_AmSp_C_vA**	gi|38197757|gb|AAR13813.1	Major ampullate spidroin-2, partial [*Argiope amoena*]	8E-10
***B. lon*_AmSp_C_vB**	gi|55274114|gb|AAV48937.1|	Dragline silk spidroin 1, partial [*Psechrus sinensis*]	0.081
***B. lon*_AmSp_C_vC**	gi|422900780|gb|AFX83566.1|	Aciniform spidroin 1, partial [Latrodectus geometricus]	2E-20
***B. lon*_AmSp_C_vD**	gi|38197749|gb|AAR13809.1|	Major ampullate spidroin-2, partial [*Argiope amoena*]	0.076
***B. lon*_AmSp_C_vE**	gi|55274104|gb|AAV48932.1|	Dragline silk spidroin 1, partial [*Cyrtophora moluccensis*]	5E-05
***B. lon*_AmSp_C_vF**	gi|55274094|gb|AAV48927.1|	Dragline silk spidroin 1, partial [*Nephila pilipes*]	3E-07
***B. lon*_CrSp_C**	gi|392997864|gb|AFM97615.1|	Fibroin 1, partial [*Hypochilus thorelli*]	0.019
***B. lon*_PySp_C**	gi|675382271|KFM75168.1|	Hypothetical protein X975_11824, partial [*Stegodyphus mimosarum*]	6E-07
***B. lon*_TuSp_C**	gi|303307770|gb|ADM14323.1|	Tubuliform spidroin 1, partial [*Agelenopsis aperta*]	7E-04
***B. lon*_AcSp_N_vA**	gi|422900780|gb|AFX83566.1|	Aciniform spidroin 1, partial [*Latrodectus geometricus*]	1E-42
***B. lon*_AcSp_N_vB**	gi|422900780|gb|AFX83566.1|	Aciniform spidroin 1, partial [*Latrodectus geometricus*]	2E-41
***B. lon*_AmSp_N_vA**	gi|115635734|emb|CAJ90517.1|	Major ampullate spidroin 1 precursor, partial [*Euprosthenops australis*]	3E-30
***B. lon*_AmSp_N_vB**	gi|87133239|gb|ABD24294.1|	Major ampullate spidroin 1, partial [*Latrodectus hesperus*]	8E-14
***B. lon*_AmSp_N_vC**	gi|115635734|emb|CAJ90517.1|	Major ampullate spidroin 1 precursor, partial [*Euprosthenops australis*]	5E-15
***B. lon*_AmSp_N_vD**	gi|193506893|gb|ACF19412.1|	Major ampullate spidroin 1B precursor, partial [*Nephila clavipes*]	9E-27
***B. lon*_AmSp_N_vE**	gi|115635734|emb|CAJ90517.1|	Major ampullate spidroin 1 precursor, partial [*Euprosthenops australis*]	1E-26
***B. lon*_AmSp_N_vF**	gi|193506893|gb|ACF19412.1|	Major ampullate spidroin 1B precursor, partial [*Nephila clavipes]*	6E-31
***B. lon*_AmSp_N_vG**	gi|193506893|gb|ACF19412.1|	Major ampullate spidroin 1B precursor, partial [*Nephila clavipes*]	2E-49
***B. lon*_Sp_N_vA**	gi|587655204|gb|AHK09765.1|	Aciniform spidroin 1 N-terminal region variant 3, partial [*Argiope argentata*]	8E-15
***B. lon*_PySp_N**	gi|675382271|gb|KFM75168.1|	Hypothetical protein X975_11824 [*Stegodyphus mimosarum*]	8E-22
***B. lon*_Sp_N_vC**	gi|422900780|gb|AFX83566.1|	Aciniform spidroin 1, partial [*Latrodectus geometricus*]	2e-23
***B. lon*_Sp_N_vB**	gi|303307752|gb|ADM14314.1|	Major ampullate spidroin 1, partial [*Kukulcania hibernalis*]	2e-10
***B. lon*_Sp_N_vD**	gi|303307750|gb|ADM14313.1|	Fibroin 1, partial [*Bothriocyrtum californicum*]	2e-11
***B. lon*_TuSp_N**	gi|303307781|gb|ADM14330.1|	Tubuliform spidroin 1, partial [*Agelenopsis aperta*]	1E-16
***D. mar*_AcSp_C**	gi|587655300|gb|AHK09813.1|	Aciniform spidroin 1 [*Argiope argentata*]	2E-08
***D. mar*_AmSp_C_vA**	gi|32815671|gb|AAP88232.1|	Major ampullate spidroin-1, partial [*Argiope amoena*]	9E-12
***D. mar*_AmSp_C_vB**	gi|32815671|gb|AAP88232.1|	Major ampullate spidroin-1, partial [*Argiope amoena*]	2E-14

***D. mar*_PySp_C**	gi|675382271|gb|KFM75168.1|	Hypothetical protein X975_11824 [*Stegodyphus mimosarum*]	1E-50
***D. mar*_TuSp_C**	gi|303307770|gb|ADM14323.1|	Tubuliform spidroin 1, partial [*Agelenopsis aperta*]	7E-17
***D. mar*_Sp_C**	gi|1263285|gb|AAC47009.1|	Fibroin-2, partial [*Araneus diadematus*]	3E-20
***D. mar*_AcSp_N**	gi|422900780|gb|AFX83566.1|	Aciniform spidroin 1, partial [*Latrodectus geometricus*]	2E-47
***D. mar*_AmSp_N_vA**	gi|193506891|gb|ACF19411.1|	Major ampullate spidroin 1A precursor, partial [*Nephila clavipes*]	2E-35
***D. mar*_AmSp_N_vB**	gi|303307772|gb|ADM14324.1|	Major ampullate spidroin, partial [*Agelenopsis aperta*]	1E-21
***D. mar*_PySp_N**	gi|675382271|gb|KFM75168.1|	Hypothetical protein X975_11824 [*Stegodyphus mimosarum*]	4E-19
***D. mar*_Sp_N**	gi|164709230|gb|ABY67420.1|	Major ampullate spidroin 1 locus 3, partial [*Latrodectus geometricus*]	1E-04
***D. mar*_TuSp_N**	gi|303307781|gb|ADM14330.1|	Tubuliform spidroin 1, partial [*Agelenopsis aperta*]	2E-17

[a] N or C in spidroin names indicate whether a contig contains the N- or C- terminal region coding sequence. Variant name (e.g. _vA) does not indicate association of N- term transcripts with C-terminal transcripts.

Table S2. Proteins identified in *Argyroneta aquatica* diving bell.

Identified Protein	Top BLAST Hit Accession	Top BLAST Hit Description	E-value
***A. aqu*_AcSp_N_vA**	gi|193506893|gb|ACF19412.1|	Tubuliform spidroin 1, partial [*Agelenopsis aperta*]	9.0E-25
***A. aqu*_PySp_C**	gi|257124471|gb|ACV41934.1|	Pyriform spidroin [*Latrodectus hesperus*]	9.0E-05
***A. aqu*_Sp_N**	gi|422900780|gb|AFX83566.1|	Aciniform spidroin 1, partial [*Latrodectus geometricus*]	2.0e-23
**DN13205_c0_g1_i1-1**	gi|33667938|gb|AAQ24546.1|	Blo t 9 allergen [*Blomia tropicalis*]	2.0E-09
**253304_1**	gi|40548521|gb|AAR87381.1|	Tropomyosin [*Neoscona nautica*]	2.0E-149
**50840_1**	gi|318087320|gb|ADV40252.1|	Hypothetical protein, partial [*Latrodectus hesperus*]	8.0E-07
**DN141454_c0_g2_i1-1**	gi|506965343|gb|AGM32062.1|	Hypothetical protein [*Coptotermes formosanus*]	6.4E-02
**242175_1**	gi|523712986|gb|AGQ56699.1|	Vitellogenin 2 [*Neoseiulus cucumeris*]	2.0E-14
**DN139557_c0_g2_i1-1**	gi|1009581138|ref|XP_015921887.1|	Apolipophorins-like [*Parasteatoda tepidariorum*]	5.0E-37
**31706_7562**	gi|2073373|dbj|BAA19844.1|	Alpha-2-macroglobulin [*Limulus sp.*]	0.0E+00
**318196_1**	gi|344178917|dbj|BAK64111.1|	Alpha-2 macroglobulin [*Hasarius adansoni*]	2.0E-49
**332195_1**	gi|671759229|gb|AII98023.1|	BLTX669 [*Nephila pilipes*]	6.0E-10
**DN138176_c0_g2_i2-1**	gi|332027696|gb|EGI67764.1|	Tubulin alpha-1 chain [*Acromyrmex echinatior*]	0.0E+00
**DN126632_c0_g5_i1-1**	gi|405952567|gb|EKC20363.1|	Hypothetical protein CGI_10006238 [*Crassostrea gigas*]	1.0E-35
**370852_1**	gi|556102150|gb|ESO90802.1|	Hypothetical protein LOTGIDRAFT_233588 [*Lottia gigantea*]	3.0E-05
**26982_11**	gi|431921509|gb|ELK18875.1|	Heat shock cognate 71 protein [*Pteropus alecto*]	4.4E+00
**DN76940_c0_g1_i1-1**	gi|492904312|ref|WP_006034718.1|	Elongation factor Tu [*Rickettsiella grylli*]	2.0E-94
**DN75073_c0_g1_i1-1**	gi|492904817|ref|WP_006035223.1|	Thioredoxin [*Rickettsiella grylli*]	7.0E-52
**DN104795_c0_g1_i1-1**	gi|499265039|ref|WP_010962432.1|	MULTISPECIES: ATP synthase subunit beta [*Wolbachia*]	0.0E+00
**21585_7**	gi|170063054|ref|XP_001866937.1|	Scavenger receptor cysteine-rich protein [*Culex quinquefasciatus*]	5.0E-67
**265412_1**	gi|241575657|ref|XP_002403224.1|	Trichohyalin, putative [*Ixodes scapularis*]	9.0E-106
**345535_1**	gi|241744740|ref|XP_002405466.1|	Alkyl hydroperoxide reductase, thiol specific antioxidant, putative [*Ixodes scapularis*]	5.0E-112
**DN131058_c3_g1_i1-1**	gi|241999408|ref|XP_002434347.1|	Glyceraldehyde 3-phosphate dehydrogenase, putative [*Ixodes scapularis*]	0.0E+00
**DN143327_c0_g2_i1-1**	gi|291225136|ref|XP_002732558.1|	Probable serine/threonine-protein kinase kinX [*Saccoglossus kowalevskii*]	1.0E-03
**DN120520_c0_g2_i1-1**	gi|291241282|ref|XP_002740542.1|	Apolipoprotein D-like [*Saccoglossus kowalevskii*]	1.0E-45
**322416_3**	gi|302783030|ref|XP_002973288.1|	Hypothetical protein SELMODRAFT_232026 [*Selaginella moellendorffii*]	1.0E-20
**31706_6984**	gi|339261586|ref|XP_003367832.1|	Putative trypsin Inhibitor like cysteine rich domain protein, partial [*Trichinella spiralis*]	1.0E-03

**281967_1**	gi|391326419|ref|XP_003737714.1|	PREDICTED: glutamate dehydrogenase, mitochondrial [*Galendromus occidentalis*]	0.0E+00
**DN32275_c0_g2_i1-1**	gi|391341738|ref|XP_003745184.1|	PREDICTED: synaptotagmin-15-like [*Metaseiulus occidentalis*]	1.0E-45
**358067_1**	gi|391342852|ref|XP_003745729.1|	PREDICTED: glucose dehydrogenase [FAD, quinone] [*Galendromus occidentalis*]	4.0E-143
**31706_8269**	gi|391342852|ref|XP_003745729.1|	PREDICTED: glucose dehydrogenase [FAD, quinone] [*Galendromus occidentalis*]	3.0E-145
**282009_1**	gi|391342852|ref|XP_003745729.1|	PREDICTED: glucose dehydrogenase [FAD, quinone] [*Galendromus occidentalis*]	7.0E-138
**DN137736_c0_g1_i1-1**	gi|499048261|ref|XP_004574680.1|	Polyserase-2-like [*Maylandia zebra*]	1.0E-30
**280834_1**	gi|556948298|ref|XP_005987015.1|	PREDICTED: low choriolytic enzyme-like isoform X2 [*Latimeria chalumnae*]	1.0E-39
**DN143934_c0_g2_i2-1**	gi|594066187|ref|XP_006057071.1|	PREDICTED: apolipoprotein L3-like [*Bubalus bubalis*]	4.0E-03
**DN133244_c0_g1_i2-1**	gi|573910962|ref|XP_006643199.1|	PREDICTED: transmembrane protease serine 9-like [*Lepisosteus oculatus*]	1.0E-43
**31706_1002**	--	--	--
**DN119698_c1_g1_i1-1**	--	--	--
**DN134830_c112_g2_i2-1**	--	--	--
**DN197674_c0_g1_i1-1**	--	--	--
**DN21975_c0_g1_i1-1**	--	--	--
**44288_1**	--	--	--
**DN152011_c3_g1_i1-1**	--	--	--
**DN152897_c7_g1_i1-1**	--	--	--
**365077_1**	--	--	--
**DN150297_c1_g5_i1-1**	--	--	--
**DN17735_c0_g1_i1-1**	--	--	--
**DN129222_c0_g1_i1-1**	--	--	--

Table S3. Summary of *de novo* transcriptome assemblies.

	Argyroneta aquatica	***Badumna longinqua***	***Desis marina***
**No. Raw Paired Reads**	57,211,947	12,104,773	112,108,725
**No. Cleaned Paired Reads**	52,277,995	7,805,324	99,597,666
**No. Contigs**	343,077	49,520	402,833
**Total Length (bp)**	209,100,116	21,859,077	263,857,902
**N50 (bp)**	805	471	892
**BUSCO**	91	66	97.5

Table S4. Sequences used in Blastx searches to identify spider silk sequences

Protein Name	Accession ID	Description	Organism
**Aciniform Spidroin**	GGRN01042810.1	D.tri_AcSp_C_vA	*Dolomedes triton*
**Aciniform Spidroin**	GGRN01034313.1	D.tri_AcSp_C_vB_contig	*Dolomedes triton*
**Aciniform Spidroin**	GGRN01039088.1	D.tri_AcSp_N_vA_contig	*Dolomedes triton*
**Aciniform Spidroin**	GGRN01024633.1	D.tri_AcSp_N_vB_contig	*Dolomedes triton*
**Aciniform Spidroin**	GGOF01004031.1	T.per_AcSp_C_contig	*Tengella perfuga*
**Aciniform Spidroin**	GGOF01043170.1	T.per_AcSp_N_contig	*Tengella perfuga*
**Aciniform Spidroin 1**	ADM35668.1	aciniform spidroin 1 [Araneus ventricosus]	*Araneus ventricosus*
**Aciniform Spidroin 1**	ADM35669.1	aciniform spidroin 1 [Argiope amoena]	*Argiope amoena*
**Aciniform Spidroin 1**	AAR83925.1	aciniform spidroin 1 [Argiope trifasciata]	*Argiope trifasciata*
**Aciniform Spidroin 1**	AFX83557.1	aciniform spidroin 1 [Latrodectus hesperus]	*Latrodectus hesperus*
**Aciniform Spidroin 1**	AFX83566.1	aciniform spidroin 1, partial [Latrodectus geometricus]	*Latrodectus geometricus*
**Aciniform Spidroin 1**	AFX83567.1	aciniform spidroin 1, partial [Latrodectus geometricus]	*Latrodectus geometricus*
**Aciniform Spidroin 1**	AFX83568.1	aciniform spidroin 1, partial [Latrodectus geometricus]	*Latrodectus geometricus*
**Aciniform Spidroin 1**	AFX83558.1	aciniform spidroin 1, partial [Latrodectus hesperus]	*Latrodectus hesperus*
**Aciniform Spidroin 1**	AFX83559.1	aciniform spidroin 1, partial [Latrodectus hesperus]	*Latrodectus hesperus*
**Aciniform Spidroin 1**	AFX83560.1	aciniform spidroin 1, partial [Latrodectus hesperus]	*Latrodectus hesperus*
**Aciniform Spidroin 1**	AFX83561.1	aciniform spidroin 1, partial [Latrodectus hesperus]	*Latrodectus hesperus*
**Aciniform Spidroin 1**	AFX83562.1	aciniform spidroin 1, partial [Latrodectus hesperus]	*Latrodectus hesperus*
**Aciniform Spidroin 1**	AFX83563.1	aciniform spidroin 1, partial [Latrodectus hesperus]	*Latrodectus hesperus*
**Aciniform Spidroin 1**	AFX83565.1	aciniform spidroin 1, partial [Latrodectus hesperus]	*Latrodectus hesperus*
**Aciniform Spidroin 1**	ADG57593.1	aciniform-like protein [Parawixia bistriata]	*Parawixia bistriata*
**Aciniform Spidroin 1**	ABD61598.1	AcSp1 [Uloborus diversus]	*Uloborus diversus*
**Aciniform Spidroin 1 -Like**	ABW24499.1	aciniform spidroin 1-like, partial [Latrodectus hesperus]	*Latrodectus hesperus*
**Aciniform Spidroin -Putative**	AZAQ01111412.1	S.m. AcSp-putative	*Stegodyphus mimosarum*
**Aggregate Gland Silk Factor 1**	AFP57565.1	aggregate gland silk factor 1, partial [Latrodectus hesperus]	*Latrodectus hesperus*
**Aggregate Gland Silk Factor 2**	AFP57562.1	aggregate gland silk factor 2 [Latrodectus hesperus]	*Latrodectus hesperus*
**Aggregate Gland Silk Factor 2-Like**	AFP57559.1	aggregate spider glue 2-like protein, partial [Latrodectus hesperus]	*Latrodectus hesperus*
**Aggregate Spidroin-1**	AMK48679.1	aggregate spidroin 1, partial [Steatoda grossa]	*Steatoda grossa*
**Aggregate Spidroin-1**	AMK48678.1	aggregate spidroin 1, partial [Parasteatoda tepidariorum]	*Parasteatoda tepidariorum*
**Aggregate Spidroin-1**	AMK48675.1	aggregate spidroin 1, partial [Argiope argentata]	*Argiope argentata*
**Aggregate Spidroin-1**	AMK48674.1	aggregate spidroin 1, partial [Araneus diadematus]	*Araneus diadematus*

**Aggregate Spidroin-1**	AMK48677.1	aggregate spidroin 1, partial [Nephila clavipes]	*Nephila clavipes*
**Aggregate Spidroin-1**	AMK48676.1	aggregate spidroin 1, partial [Latrodectus hesperus]	*Latrodectus hesperus*
**Ampullate Spidroin**	GGOF01047967.1	Tpercomp47195_c1-1_Amp_N_vA	*Tengella perfuga*
**Ampullate Spidroin**	GGOF01046653.1	Tpercomp55599_c0-1_Amp_N_vB	*Tengella perfuga*
**Ampullate Spidroin**	GGOF01039047.1	Tpercomp55839_c1-1_Amp_N_vC	*Tengella perfuga*
**Ampullate Spidroin**	GGOF01123749.1	T.per_Amp_C_vA_contig	*Tengella perfuga*
**Ampullate Spidroin**	GGOF01084018.1	T.per_Amp_C_vB_contig	*Tengella perfuga*
**Ampullate Spidroin**	GGOF01083989.1	T.per_Amp_C_vC_contig	*Tengella perfuga*
**Ampullate Spidroin**	GGRN01028718.1	D.tri_Amp_C_vA_contig	*Dolomedes triton*
**Ampullate Spidroin**	GGRN01042502.1	D.tri_Amp_vB_contig	*Dolomedes triton*
**Ampullate Spidroin**	GGRN01017618.1	12534_1_Amp_C_vC	*Dolomedes triton*
**Ampullate Spidroin**	GGRN01023437.1	3010_1_Amp_C_vD	*Dolomedes triton*
**Ampullate Spidroin**	GGRN01022108.1	D.tri_Amp_N_vA_contig	*Dolomedes triton*
**Ampullate Spidroin**	GGRN01004068.1	DolMalecomp6778_c4-1_Amp_N_vB	*Dolomedes triton*
**Ampullate Spidroin**	GGRN01039044.1	196_1_Amp_N_vC	*Dolomedes triton*
**Ampullate Spidroin**	GGRN01040476.1	8251_1_Amp_N_vD	*Dolomedes triton*
**Ampullate Spidroin**	GGRN01013787.1	DolFecomp3105_c0-1_Amp_N_vE	*Dolomedes triton*
**Aqueous Glue Droplet Peptide-1**	ABO09798.1	aqueous glue droplet peptide [Latrodectus hesperus]	*Latrodectus hesperus*
**Aqueous Glue Droplet Peptide-2**	ABO09799.1	aqueous glue droplet peptide [Latrodectus hesperus]	*Latrodectus hesperus*
**Cribellar Spidroin**	GGOF01121319.1	T.per_CrSp_C_contig	*Tengella perfuga*
**Dragline Silk Fibroin 1**	P46802.1	Dragline silk fibroin 1 [Araneus bicentenarius]	*Araneus bicentenarius*
**Dragline Silk Fibroin 1**	AAV48932.1	dragline silk spidroin 1 [Cyrtophora moluccensis]	*Cyrtophora moluccensis*
**Dragline Silk Fibroin 1**	AAV48934.1	dragline silk spidroin 1 [Cyrtophora moluccensis]	*Cyrtophora moluccensis*
**Dragline Silk Fibroin 1**	AAV48935.1	dragline silk spidroin 1 [Cyrtophora moluccensis]	*Cyrtophora moluccensis*
**Dragline Silk Fibroin 1**	AAV48951.1	dragline silk spidroin 1 [Cyrtophora moluccensis]	*Cyrtophora moluccensis*
**Dragline Silk Fibroin 1**	AAV48940.1	dragline silk spidroin 1 [Macrothele holsti]	*Macrothele holsti*
**Dragline Silk Fibroin 1**	AAV48920.1	dragline silk spidroin 1 [Nephila pilipes]	*Nephila pilipes*
**Dragline Silk Fibroin 1**	AAV48921.1	dragline silk spidroin 1 [Nephila pilipes]	*Nephila pilipes*
**Dragline Silk Fibroin 1**	AAV48922.1	dragline silk spidroin 1 [Nephila pilipes]	*Nephila pilipes*
**Dragline Silk Fibroin 1**	AAV48925.1	dragline silk spidroin 1 [Nephila pilipes]	*Nephila pilipes*
**Dragline Silk Fibroin 1**	AAV48926.1	dragline silk spidroin 1 [Nephila pilipes]	*Nephila pilipes*
**Dragline Silk Fibroin 1**	AAV48927.1	dragline silk spidroin 1 [Nephila pilipes]	*Nephila pilipes*
**Dragline Silk Fibroin 1**	AAV48928.1	dragline silk spidroin 1 [Nephila pilipes]	*Nephila pilipes*
**Dragline Silk Fibroin 1**	AAV48941.1	dragline silk spidroin 1 [Nephila pilipes]	*Nephila pilipes*
**Dragline Silk Fibroin 1**	AAV48942.1	dragline silk spidroin 1 [Nephila pilipes]	*Nephila pilipes*

**Dragline Silk Fibroin 1**	AAV48943.1	dragline silk spidroin 1 [Nephila pilipes]	*Nephila pilipes*
**Dragline Silk Fibroin 1**	AAV48944.1	dragline silk spidroin 1 [Nephila pilipes]	*Nephila pilipes*
**Dragline Silk Fibroin 1**	AAV48945.1	dragline silk spidroin 1 [Nephila pilipes]	*Nephila pilipes*
**Dragline Silk Fibroin 1**	AAV48947.1	dragline silk spidroin 1 [Nephila pilipes]	*Nephila pilipes*
**Dragline Silk Fibroin 1**	AAV48948.1	dragline silk spidroin 1 [Nephila pilipes]	*Nephila pilipes*
**Dragline Silk Fibroin 1**	AAV48929.1	dragline silk spidroin 1 [Octonoba varians]	*Octonoba varians*
**Dragline Silk Fibroin 1**	AAV48938.1	dragline silk spidroin 1 [Psechrus sinensis]	*Psechrus sinensis*
**Dragline Silk Fibroin 1**	AAV48939.1	dragline silk spidroin 1, partial [Psechrus sinensis]	*Psechrus sinensis*
**Dragline Silk Fibroin 1**	P19837.3	RecName: Full=Spidroin-1; AltName: Full=Dragline silk fibroin 1, partial [Nephila clavipes]	*Nephila clavipes*
**Dragline Silk Fibroin 2**	P46804.1	RecName: Full=Spidroin-2; AltName: Full=Dragline silk fibroin 2, partial [Nephila clavipes]	*Nephila clavipes*
**Dragline Silk Protein**	AAL32375.1	dragline silk protein [Nephila clavipes]	*Nephila clavipes*
**Dragline Silk Protein Spidroin 1**	AAC04504.1	dragline silk protein spidroin 1 [Nephila clavipes]	*Nephila clavipes*
**Dragline Silk Protein Spidroin 2**	AAL32472.1	dragline silk protein spidroin 2 [Nephila clavata]	*Nephila clavata*
**Egg Case Fibroin**	AAZ15706.1	egg case fibroin [Latrodectus hesperus]	*Latrodectus hesperus*
**Egg Case Fibroin**	ADV40181.1	egg case fibroin [Latrodectus hesperus]	*Latrodectus hesperus*
**Egg Case Fibroin-Like Protein 1**	AFM97608.1	egg case protein-like protein 1 [Liphistius malayanus]	*Liphistius malayanus*
**Egg Case Fibroin-Like Protein 2**	AFM97609.1	egg case protein-like protein 2 [Liphistius malayanus]	*Liphistius malayanus*
**Egg Case Fibroin-Like Protein 3**	AFM97610.1	egg case protein-like protein 3 [Liphistius malayanus]	*Liphistius malayanus*
**Egg Case Fibroin-Like Protein 4**	AFM97611.1	egg case protein-like protein 4 [Liphistius malayanus]	*Liphistius malayanus*
**Egg Case Fibroin-Like Protein 5**	AFM97612.1	egg case protein-like protein 5, partial [Liphistius malayanus]	*Liphistius malayanus*
**Egg Case Fibroin-Like Protein 6**	AFM97613.1	egg case protein-like protein 6 [Liphistius malayanus]	*Liphistius malayanus*
**Egg Case Silk Protein 1**	BAE86855.1	egg case silk protein 1 [Argiope bruennichi]	*Argiope bruennichi*
**Egg Case Silk Protein 1**	AAX92677.1	egg case silk protein-1 [Latrodectus hesperus]	*Latrodectus hesperus*
**Egg Case Silk Protein 2**	BAE86856.1	egg case silk protein 2 [Argiope bruennichi]	*Argiope bruennichi*
**Egg Case Silk Protein 2**	ABC68105.1	egg case silk protein 2 [Latrodectus hesperus]	*Latrodectus hesperus*
**Fibroin 1**	ABW80563.1	fibroin 1 [Aptostichus sp. AS217]	*Aptostichus sp. AS217*
**Fibroin 1**	ABW80565.1	fibroin 1 [Bothriocyrtum californicum]	*Bothriocyrtum californicum*
**Fibroin 1**	ADM14313.1	fibroin 1 [Bothriocyrtum californicum]	*Bothriocyrtum californicum*
**Fibroin 1**	AAK30598.1	fibroin 1 [Dolomedes tenebrosus]	*Dolomedes tenebrosus*
**Fibroin 1**	AAK30600.1	fibroin 1 [Euagrus chisoseus]	*Euagrus chisoseus*
**Fibroin 1**	ABW80568.1	fibroin 1 [Euagrus chisoseus]	*Euagrus chisoseus*
**Fibroin 1**	AAK30610.1	fibroin 1 [Plectreurys tristis]	*Plectreurys tristis*
**Fibroin 1**	ABW80562.1	fibroin 1, partial [Aliatypus gulosus]	*Aliatypus gulosus*
**Fibroin 1**	AFM97617.1	fibroin 1, partial [Aphonopelma seemanni]	*Aphonopelma seemanni*

**Fibroin 1**	AFM97622.1	fibroin 1, partial [Atypoides riversi]	*Atypoides riversi*
**Fibroin 1**	AFM97625.1	fibroin 1, partial [Hexura picea]	*Hexura picea*
**Fibroin 1**	AFM97615.1	fibroin 1, partial [Hypochilus thorelli]	*Hypochilus thorelli*
**Fibroin 1**	AFM97627.1	fibroin 1, partial [Megahexura fulva]	*Megahexura fulva*
**Fibroin 1**	AFM97620.1	fibroin 1, partial [Poecilotheria regalis]	*Poecilotheria regalis*
**Fibroin 1**	AIU80193.1	fibroin 1, partial [Scytodes thoracica]	*Scytodes thoracica*
**Fibroin 1a**	ABD61591.1	fibroin 1a [Deinopis spinosa]	*Deinopis spinosa*
**Fibroin 1b**	ABD61592.1	fibroin 1b [Deinopis spinosa]	*Deinopis spinosa*
**Fibroin 2**	ABW80564.1	fibroin 2 [Aptostichus sp. AS220]	*Aptostichus sp. AS220*
**Fibroin 2**	ABD61588.1	fibroin 2 [Deinopis spinosa]	*Deinopis spinosa*
**Fibroin 2**	AAK30599.1	fibroin 2 [Dolomedes tenebrosus]	*Dolomedes tenebrosus*
**Fibroin 2**	AAK30611.1	fibroin 2 [Plectreurys tristis]	*Plectreurys tristis*
**Fibroin 2**	AFM97618.1	fibroin 2, partial [Aphonopelma seemanni]	*Aphonopelma seemanni*
**Fibroin 2**	AFM97623.1	fibroin 2, partial [Atypoides riversi]	*Atypoides riversi*
**Fibroin 2**	AFM97616.1	fibroin 2, partial [Hypochilus thorelli]	*Hypochilus thorelli*
**Fibroin 2**	AFM97621.1	fibroin 2, partial [Poecilotheria regalis]	*Poecilotheria regalis*
**Fibroin 2**	AIU80194.1	fibroin 2, partial [Scytodes thoracica]	*Scytodes thoracica*
**Fibroin 3**	AFM97619.1	fibroin 3, partial [Aphonopelma seemanni]	*Aphonopelma seemanni*
**Fibroin 4**	AAK30613.1	fibroin 4 [Plectreurys tristis]	*Plectreurys tristis*
**Fibroin-1**	AAC47008.1	fibroin-1, partial [Araneus diadematus]	*Araneus diadematus*
**Fibroin-2**	AAC47009.1	fibroin-2, partial [Araneus diadematus]	*Araneus diadematus*
**Fibroin-3**	AAC47010.1	fibroin-3, partial [Araneus diadematus]	*Araneus diadematus*
**Fibroin-4**	AAC47011.1	fibroin-4, partial [Araneus diadematus]	*Araneus diadematus*
**Flagelliform Silk Protein**	ABD61590.1	Flag [Deinopis spinosa]	*Deinopis spinosa*
**Flagelliform Silk Protein**	AAK30594.1	flagelliform silk protein [Argiope trifasciata]	*Argiope trifasciata*
**Flagelliform Silk Protein**	AAC38847.1	flagelliform silk protein [Nephila clavipes]	*Nephila clavipes*
**Flagelliform Silk Protein**	AAF36092.1	flagelliform silk protein, partial [Nephila inaurata madagascariensis]	*Nephila inaurata madagascariensis*
**Flagelliform Silk Protein-1**	AAT36347.1	flagelliform silk protein-1 [Araneus ventricosus]	*Araneus ventricosus*
**Flagelliform Silk Protein-Like**	ABR37273.1	flagelliform spidroin-like protein, partial [Nephilengys cruentata]	*Nephilengys cruentata*
**Major Ampullate Gland Dragline Silk Protein 2**	AAN85281.1	major ampullate gland dragline silk protein-2 [Araneus ventricosus]	*Araneus ventricosus*
**Major Ampullate Silk Protein-Putative-a**	AZAQ01054438.1	S.m.MaSp-putative-a	*Stegodyphus mimosarum*
**Major Ampullate Silk Protein-Putative-b**	AZAQ01171519.1	S.m.MaSp-putative-b	*Stegodyphus mimosarum*
**Major Ampullate Silk Protein-Putative-c**	AZAQ01121857.1	S.m.MaSp-putative-c	*Stegodyphus mimosarum*
**Major Ampullate Silk Protein-Putative-d**	AZAQ01026555.1	S.m.MaSp-putative-d	*Stegodyphus mimosarum*

**Major Ampullate Silk Protein-Putative-e**	AZAQ01086913.1	S.m.MaSp-putative-e	*Stegodyphus mimosarum*
**Major Ampullate Silk Protein-Putative-f**	AZAQ01026550.1	S.m.MaSp-putative-f	*Stegodyphus mimosarum*
**Major Ampullate Silk Protein-Putative-g**	AZAQ01005949.1	S.m.MaSp-putative-g	*Stegodyphus mimosarum*
**Major Ampullate Silk Protein-Putative-h**	AZAQ01026555.1	S.m.MaSp-putative-h	*Stegodyphus mimosarum*
**Major Ampullate Silk Protein-Putative-i**	AZAQ01107911.1	S.m.MaSp-putative-i	*Stegodyphus mimosarum*
**Major Ampullate Silk Protein-Putative-j**	AZAQ01107913.1	S.m.MaSp-putative-j	*Stegodyphus mimosarum*
**Major Ampullate Silk Protein-Putative-k**	AZAQ01108596.1	S.m.MaSp-putative-k	*Stegodyphus mimosarum*
**Major Ampullate Spidroin**	AAT08436.1	major ampullate spidroin [Agelenopsis aperta]	*Agelenopsis aperta*
**Major Ampullate Spidroin**	ADM14324.1	major ampullate spidroin [Agelenopsis aperta]	*Agelenopsis aperta*
**Major Ampullate Spidroin**	ADM14325.1	major ampullate spidroin [Agelenopsis aperta]	*Agelenopsis aperta*
**Major Ampullate Spidroin**	ADM14315.1	major ampullate spidroin [Diguetia canities]	*Diguetia canities*
**Major Ampullate Spidroin**	ADM14316.1	major ampullate spidroin [Diguetia canities]	*Diguetia canities*
**Major Ampullate Spidroin 1**	ADO78764.1	major ampullate spidroin 1, partial [Latrodectus mactans]	*Latrodectus mactans*
**Major Ampullate Spidroin 1**	ABR68856.1	major ampullate spidroin 1 [Latrodectus hesperus].	*Latrodectus hesperus*
**Major Ampullate Spidroin 1**	ABR68857.1	major ampullate spidroin 1, partial [Latrodectus hesperus]	*Latrodectus hesperus*
**Major Ampullate Spidroin 1**	ABC72644.1	major ampullate fibroin 1 [Nephila antipodiana]	*Nephila antipodiana*
**Major Ampullate Spidroin 1**	AFN54362.1	major ampullate silk protein 1, partial [Argiope bruennichi]	*Argiope bruennichi*
**Major Ampullate Spidroin 1**	AAK30591.1	major ampullate spidroin 1 [Argiope aurantia]	*Argiope aurantia*
**Major Ampullate Spidroin 1**	CAJ00428.1	major ampullate spidroin 1 [Euprosthenops australis]	*Euprosthenops australis*
**Major Ampullate Spidroin 1**	CAM32251.1	major ampullate spidroin 1 [Euprosthenops australis]	*Euprosthenops australis*
**Major Ampullate Spidroin 1**	CAM32252.1	major ampullate spidroin 1 [Euprosthenops australis]	*Euprosthenops australis*
**Major Ampullate Spidroin 1**	CAM32253.1	major ampullate spidroin 1 [Euprosthenops australis]	*Euprosthenops australis*
**Major Ampullate Spidroin 1**	CAM32254.1	major ampullate spidroin 1 [Euprosthenops australis]	*Euprosthenops australis*
**Major Ampullate Spidroin 1**	CAM32255.1	major ampullate spidroin 1 [Euprosthenops australis]	*Euprosthenops australis*
**Major Ampullate Spidroin 1**	CAM32256.1	major ampullate spidroin 1 [Euprosthenops australis]	*Euprosthenops australis*
**Major Ampullate Spidroin 1**	CAM32257.1	major ampullate spidroin 1 [Euprosthenops australis]	*Euprosthenops australis*
**Major Ampullate Spidroin 1**	CAM32258.1	major ampullate spidroin 1 [Euprosthenops australis]	*Euprosthenops australis*
**Major Ampullate Spidroin 1**	CAM32259.1	major ampullate spidroin 1 [Euprosthenops australis]	*Euprosthenops australis*
**Major Ampullate Spidroin 1**	CAM32260.1	major ampullate spidroin 1 [Euprosthenops australis]	*Euprosthenops australis*
**Major Ampullate Spidroin 1**	CAM32261.1	major ampullate spidroin 1 [Euprosthenops australis]	*Euprosthenops australis*
**Major Ampullate Spidroin 1**	CAM32262.1	major ampullate spidroin 1 [Euprosthenops australis]	*Euprosthenops australis*
**Major Ampullate Spidroin 1**	CAM32263.1	major ampullate spidroin 1 [Euprosthenops australis]	*Euprosthenops australis*
**Major Ampullate Spidroin 1**	CAM32264.1	major ampullate spidroin 1 [Euprosthenops australis]	*Euprosthenops australis*
**Major Ampullate Spidroin 1**	CAM32265.1	major ampullate spidroin 1 [Euprosthenops australis]	*Euprosthenops australis*

**Major Ampullate Spidroin 1**	CAM32267.1	major ampullate spidroin 1 [Euprosthenops australis]	*Euprosthenops australis*
**Major Ampullate Spidroin 1**	CAM32268.1	major ampullate spidroin 1 [Euprosthenops australis]	*Euprosthenops australis*
**Major Ampullate Spidroin 1**	CAM32269.1	major ampullate spidroin 1 [Euprosthenops australis]	*Euprosthenops australis*
**Major Ampullate Spidroin 1**	CAM32270.1	major ampullate spidroin 1 [Euprosthenops australis]	*Euprosthenops australis*
**Major Ampullate Spidroin 1**	CAM32271.1	major ampullate spidroin 1 [Euprosthenops australis]	*Euprosthenops australis*
**Major Ampullate Spidroin 1**	AAT08433.1	major ampullate spidroin 1 [Kukulcania hibernalis]	*Kukulcania hibernalis*
**Major Ampullate Spidroin 1**	ADM14314.1	major ampullate spidroin 1 [Kukulcania hibernalis]	*Kukulcania hibernalis*
**Major Ampullate Spidroin 1**	AAY28935.1	major ampullate spidroin 1 [Latrodectus hesperus]	*Latrodectus hesperus*
**Major Ampullate Spidroin 1**	ABD66602.1	major ampullate spidroin 1 [Latrodectus hesperus]	*Latrodectus hesperus*
**Major Ampullate Spidroin 1**	ABR68856.1	major ampullate spidroin 1 [Latrodectus hesperus]	*Latrodectus hesperus*
**Major Ampullate Spidroin 1**	ABR68857.1	major ampullate spidroin 1 [Latrodectus hesperus]	*Latrodectus hesperus*
**Major Ampullate Spidroin 1**	AAT75308.1	major ampullate spidroin 1 [Nephila clavipes]	*Nephila clavipes*
**Major Ampullate Spidroin 1**	AAT75309.1	major ampullate spidroin 1 [Nephila clavipes]	*Nephila clavipes*
**Major Ampullate Spidroin 1**	AAT75310.1	major ampullate spidroin 1 [Nephila clavipes]	*Nephila clavipes*
**Major Ampullate Spidroin 1**	AAT75311.1	major ampullate spidroin 1 [Nephila clavipes]	*Nephila clavipes*
**Major Ampullate Spidroin 1**	AAT75312.1	major ampullate spidroin 1 [Nephila clavipes]	*Nephila clavipes*
**Major Ampullate Spidroin 1**	ACC77633.1	major ampullate spidroin 1 [Nephila clavipes]	*Nephila clavipes*
**Major Ampullate Spidroin 1**	AAK30606.1	major ampullate spidroin 1 [Nephila inaurata madagascariensis]	*Nephila inaurata madagascariensis*
**Major Ampullate Spidroin 1**	AAK30608.1	major ampullate spidroin 1 [Nephila senegalensis]	*Nephila senegalensis*
**Major Ampullate Spidroin 1**	AAK30614.1	major ampullate spidroin 1 [Tetragnatha kauaiensis]	*Tetragnatha kauaiensis*
**Major Ampullate Spidroin 1**	AAK30615.1	major ampullate spidroin 1 [Tetragnatha versicolor]	*Tetragnatha versicolor*
**Major Ampullate Spidroin 1**	AEV46833.2	major ampullate spidroin 1 silk protein, partial [Araneus ventricosus]	*Araneus ventricosus*
**Major Ampullate Spidroin 1**	AAP88232.1	major ampullate spidroin-1 [Argiope amoena]	*Argiope amoena*
**Major Ampullate Spidroin 1**	ABD61596.1	MaSp1 [Uloborus diversus]	*Uloborus diversus*
**Major Ampullate Spidroin 1, Locus 1**	ABY67402.1	major ampullate spidroin 1 locus 1 [Latrodectus hesperus]	*Latrodectus hesperus*
**Major Ampullate Spidroin 1, Locus 1**	ABY67403.1	major ampullate spidroin 1 locus 1 [Latrodectus hesperus]	*Latrodectus hesperus*
**Major Ampullate Spidroin 1, Locus 1**	ABY67412.1	major ampullate spidroin 1 locus 1 [Latrodectus hesperus]	*Latrodectus hesperus*
**Major Ampullate Spidroin 1, Locus 1**	ABY67413.1	major ampullate spidroin 1 locus 1 [Latrodectus hesperus]	*Latrodectus hesperus*
**Major Ampullate Spidroin 1, Locus 1**	ABY67414.1	major ampullate spidroin 1 locus 1 [Latrodectus hesperus]	*Latrodectus hesperus*
**Major Ampullate Spidroin 1, Locus 1**	ABY67415.1	major ampullate spidroin 1 locus 1 [Latrodectus hesperus]	*Latrodectus hesperus*
**Major Ampullate Spidroin 1, Locus 1**	ABY67418.1	major ampullate spidroin 1 locus 1 [Latrodectus hesperus]	*Latrodectus hesperus*

**Major Ampullate Spidroin 1, Locus 1**	ABY67421.1	major ampullate spidroin 1 locus 1 [Latrodectus hesperus]	*Latrodectus hesperus*
**Major Ampullate Spidroin 1, Locus 1**	ABY67423.1	major ampullate spidroin 1 locus 1 [Latrodectus hesperus]	*Latrodectus hesperus*
**Major Ampullate Spidroin 1, Locus 2**	ABY67406.1	major ampullate spidroin 1 locus 2 [Latrodectus hesperus]	*Latrodectus hesperus*
**Major Ampullate Spidroin 1, Locus 2**	ABY67407.1	major ampullate spidroin 1 locus 2 [Latrodectus hesperus]	*Latrodectus hesperus*
**Major Ampullate Spidroin 1, Locus 2**	ABY67410.1	major ampullate spidroin 1 locus 2 [Latrodectus hesperus]	*Latrodectus hesperus*
**Major Ampullate Spidroin 1, Locus 2**	ABY67411.1	major ampullate spidroin 1 locus 2 [Latrodectus hesperus]	*Latrodectus hesperus*
**Major Ampullate Spidroin 1, Locus 2**	ABY67422.1	major ampullate spidroin 1 locus 2 [Latrodectus hesperus]	*Latrodectus hesperus*
**Major Ampullate Spidroin 1, Locus 2**	ABY67424.1	major ampullate spidroin 1 locus 2 [Latrodectus hesperus]	*Latrodectus hesperus*
**Major Ampullate Spidroin 1, Locus 2**	ABY67425.1	major ampullate spidroin 1 locus 2 [Latrodectus hesperus]	*Latrodectus hesperus*
**Major Ampullate Spidroin 1, Locus 3**	ABY67420.1	major ampullate spidroin 1 locus 3 [Latrodectus geometricus]	*Latrodectus geometricus*
**Major Ampullate Spidroin 1, Locus 3**	ABY67400.1	major ampullate spidroin 1 locus 3 [Latrodectus hesperus]	*Latrodectus hesperus*
**Major Ampullate Spidroin 1, Locus 3**	ABY67401.1	major ampullate spidroin 1 locus 3 [Latrodectus hesperus]	*Latrodectus hesperus*
**Major Ampullate Spidroin 1, Locus 3**	ABY67404.1	major ampullate spidroin 1 locus 3 [Latrodectus hesperus]	*Latrodectus hesperus*
**Major Ampullate Spidroin 1, Locus 3**	ABY67405.1	major ampullate spidroin 1 locus 3 [Latrodectus hesperus]	*Latrodectus hesperus*
**Major Ampullate Spidroin 1, Locus 3**	ABY67419.1	major ampullate spidroin 1 locus 3 [Latrodectus hesperus]	*Latrodectus hesperus*
**Major Ampullate Spidroin 1, Precursor**	CAJ90517.1	major ampullate spidroin 1 precursor [Euprosthenops australis]	*Euprosthenops australis*
**Major Ampullate Spidroin 1, Variant 1, Locus 1**	ABY67426.1	major ampullate spidroin 1 variant 1 locus 1 [Latrodectus geometricus]	*Latrodectus geometricus*
**Major Ampullate Spidroin 1, Variant 1, Locus 2**	ABY67428.1	major ampullate spidroin 1 variant 1 locus 2 [Latrodectus geometricus]	*Latrodectus geometricus*
**Major Ampullate Spidroin 1, Variant 2, Locus 1**	ABY67427.1	major ampullate spidroin 1 variant 2 locus 1 [Latrodectus geometricus]	*Latrodectus geometricus*
**Major Ampullate Spidroin 1, Variant 2, Locus 2**	ABY67429.1	major ampullate spidroin 1 variant 2 locus 2, [Latrodectus geometricus]	*Latrodectus geometricus*
**Major Ampullate Spidroin 1A, Precursor**	ACF19411.1	major ampullate spidroin 1A precursor [Nephila clavipes]	*Nephila clavipes*
**Major Ampullate Spidroin 1B, Precursor**	ACF19412.1	major ampullate spidroin 1B precursor [Nephila clavipes]	*Nephila clavipes*
**Major Ampullate Spidroin 1-Like**	AAZ15320.1	major ampullate spidroin 1-like, partial [Latrodectus geometricus]	*Latrodectus geometricus*
**Major Ampullate Spidroin 1-Like**	AAZ15321.1	major ampullate spidroin 1-like, partial [Latrodectus geometricus]	*Latrodectus geometricus*

**Major Ampullate Spidroin 1-Like**	AAK30595.1	major ampullate spidroin 1, partial [Argiope trifasciata]	*Argiope trifasciata*
**Major Ampullate Spidroin 1-Like**	AAK30602.1	major ampullate spidroin 1, partial [Latrodectus geometricus]	*Latrodectus geometricus*
**Major Ampullate Spidroin 1-Like**	ABD24294.1	major ampullate spidroin 1, partial [Latrodectus hesperus]	*Latrodectus hesperus*
**Major Ampullate Spidroin 1-Like**	ADE74592.1	major ampullate spidroin 1, partial [Peucetia viridans]	*Peucetia viridans*
**Major Ampullate Spidroin 1-Like**	ADG57596.1	MaSp1-like protein [Parawixia bistriata]	*Parawixia bistriata*
**Major Ampullate Spidroin 2**	AFN54363.1	major ampullate silk protein 2 [Argiope bruennichi]	*Argiope bruennichi*
**Major Ampullate Spidroin 2**	AAK30592.1	major ampullate spidroin 2 [Argiope aurantia]	*Argiope aurantia*
**Major Ampullate Spidroin 2**	AAK30596.1	major ampullate spidroin 2 [Argiope trifasciata]	*Argiope trifasciata*
**Major Ampullate Spidroin 2**	ADM14319.1	major ampullate spidroin 2 [Deinopis spinosa]	*Deinopis spinosa*
**Major Ampullate Spidroin 2**	CAM32249.1	major ampullate spidroin 2 [Euprosthenops australis]	*Euprosthenops australis*
**Major Ampullate Spidroin 2**	CAM32272.1	major ampullate spidroin 2 [Euprosthenops australis]	*Euprosthenops australis*
**Major Ampullate Spidroin 2**	AAK30601.1	major ampullate spidroin 2 [Gasteracantha cancriformis]	*Gasteracantha cancriformis*
**Major Ampullate Spidroin 2**	AAK30603.1	major ampullate spidroin 2 [Latrodectus geometricus]	*Latrodectus geometricus*
**Major Ampullate Spidroin 2**	AAK30604.1	major ampullate spidroin 2 [Latrodectus geometricus]	*Latrodectus geometricus*
**Major Ampullate Spidroin 2**	ABY67417.1	major ampullate spidroin 2 [Latrodectus geometricus]	*Latrodectus geometricus*
**Major Ampullate Spidroin 2**	AAY28936.1	major ampullate spidroin 2 [Latrodectus hesperus]	*Latrodectus hesperus*
**Major Ampullate Spidroin 2**	ABD66603.1	major ampullate spidroin 2 [Latrodectus hesperus]	*Latrodectus hesperus*
**Major Ampullate Spidroin 2**	ABR68855.1	major ampullate spidroin 2 [Latrodectus hesperus]	*Latrodectus hesperus*
**Major Ampullate Spidroin 2**	ABR68858.1	major ampullate spidroin 2 [Latrodectus hesperus]	*Latrodectus hesperus*
**Major Ampullate Spidroin 2**	ABY67408.1	major ampullate spidroin 2 [Latrodectus hesperus]	*Latrodectus hesperus*
**Major Ampullate Spidroin 2**	ABY67409.1	major ampullate spidroin 2 [Latrodectus hesperus]	*Latrodectus hesperus*
**Major Ampullate Spidroin 2**	ABY67416.1	major ampullate spidroin 2 [Latrodectus hesperus]	*Latrodectus hesperus*
**Major Ampullate Spidroin 2**	AAT75313.1	major ampullate spidroin 2 [Nephila clavipes]	*Nephila clavipes*
**Major Ampullate Spidroin 2**	AAT75314.1	major ampullate spidroin 2 [Nephila clavipes]	*Nephila clavipes*
**Major Ampullate Spidroin 2**	AAT75315.1	major ampullate spidroin 2 [Nephila clavipes]	*Nephila clavipes*
**Major Ampullate Spidroin 2**	AAT75316.1	major ampullate spidroin 2 [Nephila clavipes]	*Nephila clavipes*
**Major Ampullate Spidroin 2**	AAT75317.1	major ampullate spidroin 2 [Nephila clavipes]	*Nephila clavipes*
**Major Ampullate Spidroin 2**	AAK30607.1	major ampullate spidroin 2 [Nephila inaurata madagascariensis]	*Nephila inaurata madagascariensis*
**Major Ampullate Spidroin 2**	AAK30609.1	major ampullate spidroin 2 [Nephila senegalensis]	*Nephila senegalensis*
**Major Ampullate Spidroin 2**	ACF19413.1	major ampullate spidroin 2 precursor [Nephila clavipes]	*Nephila clavipes*
**Major Ampullate Spidroin 2**	AAZ15371.1	major ampullate spidroin 2, partial [Argiope trifasciata]	*Argiope trifasciata*

**Major Ampullate Spidroin 2**	AAZ15372.1	major ampullate spidroin 2, partial [Argiope trifasciata]	*Argiope trifasciata*
**Major Ampullate Spidroin 2**	ABD24295.1	major ampullate spidroin 2, partial [Latrodectus hesperus]	*Latrodectus hesperus*
**Major Ampullate Spidroin 2**	AAR13808.1	major ampullate spidroin-2 [Argiope amoena]	*Argiope amoena*
**Major Ampullate Spidroin 2**	AAR13809.1	major ampullate spidroin-2 [Argiope amoena]	*Argiope amoena*
**Major Ampullate Spidroin 2**	AAR13810.1	major ampullate spidroin-2 [Argiope amoena]	*Argiope amoena*
**Major Ampullate Spidroin 2**	AAR13811.1	major ampullate spidroin-2 [Argiope amoena]	*Argiope amoena*
**Major Ampullate Spidroin 2**	AAR13812.1	major ampullate spidroin-2 [Argiope amoena]	*Argiope amoena*
**Major Ampullate Spidroin 2**	AAR13813.1	major ampullate spidroin-2 [Argiope amoena]	*Argiope amoena*
**Major Ampullate Spidroin 2**	AAR13814.1	major ampullate spidroin-2 [Argiope amoena]	*Argiope amoena*
**Major Ampullate Spidroin 2**	ABD61599.1	MaSp2 [Uloborus diversus]	*Uloborus diversus*
**Major Ampullate Spidroin 2**	ABD61600.1	MaSp2 [Uloborus diversus]	*Uloborus diversus*
**Major Ampullate Spidroin 2-1**	AAT08434.1	major ampullate spidroin 2-1 [Kukulcania hibernalis]	*Kukulcania hibernalis*
**Major Ampullate Spidroin 2-2**	AAT08435.1	major ampullate spidroin 2-2 [Kukulcania hibernalis]	*Kukulcania hibernalis*
**Major Ampullate Spidroin 2a**	ABD61593.1	MaSp2a [Deinopis spinosa]	*Deinopis spinosa*
**Major Ampullate Spidroin 2b**	ABD61594.1	MaSp2b [Deinopis spinosa]	*Deinopis spinosa*
**Major Ampullate Spidroin 2-Like**	AAZ15322.1	major ampullate spidroin 2-like [Nephila inaurata madagascariensis]	*Nephila inaurata madagascariensis*
**Major Ampullate Spidroin 2-Like**	AAK30597.1	major ampullate spidroin 2-like protein [Argiope trifasciata]	*Argiope trifasciata*
**Major Ampullate Spidroin 2-Like**	AAK30605.1	major ampullate spidroin 2-like protein [Nephila inaurata madagascariensis]	*Nephila inaurata madagascariensis*
**Major Ampullate Spidroin 2-Like**	ADG57597.1	MaSp2-like protein, partial [Parawixia bistriata]	*Parawixia bistriata*
**Major Ampullate Spidroin 3**	AAT08432.1	major ampullate spidroin 3 [Kukulcania hibernalis]	*Kukulcania hibernalis*
**Major Ampullate Spidroin-Like**	CAM32250.1	major ampullate spidroin-like [Euprosthenops australis]	*Euprosthenops australis*
**Major Ampullate Spidroin-Like**	ADM14317.1	major ampullate spidroin-like protein [Diguetia canities]	*Diguetia canities*
**Major Ampullate Spidroin-Like**	ADM14318.1	major ampullate spidroin-like protein [Diguetia canities]	*Diguetia canities*
**Major Ampullate Spidroin-Like**	AAV91960.1	major ampullate spidroin-like protein [Latrodectus geometricus]	*Latrodectus geometricus*
**Major Ampullate Spidroin-Like**	ABR37275.1	major ampullate spidroin-like protein [Nephilengys cruentata]	*Nephilengys cruentata*
**Minor Ampullate Silk Protein**	AAC14590.1	minor ampullate silk protein [Nephila clavipes]	*Nephila clavipes*
**Minor Ampullate Silk Protein**	AF027736.1	minor ampullate silk protein [Nephila clavipes]	*Nephila clavipes*
**Minor Ampullate Silk Protein**	AFV31615.1	minor ampullate spidroin [Araneus ventricosus]	*Araneus ventricosus*
**Minor Ampullate Silk Protein**	ADM14321.1	minor ampullate spidroin [Latrodectus hesperus]	*Latrodectus hesperus*
**Minor Ampullate Silk Protein**	ADM14322.1	minor ampullate spidroin [Latrodectus hesperus]	*Latrodectus hesperus*
**Minor Ampullate Silk Protein**	ADM14320.1	minor ampullate spidroin [Metepeira grandiosa]	*Metepeira grandiosa*

**Minor Ampullate Silk Protein**	ADM14328.1	minor ampullate spidroin [Metepeira grandiosa]	*Metepeira grandiosa*
**Minor Ampullate Silk Protein**	ADM14329.1	minor ampullate spidroin [Metepeira grandiosa]	*Metepeira grandiosa*
**Minor Ampullate Silk Protein**	ADM14326.1	minor ampullate spidroin [Uloborus diversus]	*Uloborus diversus*
**Minor Ampullate Silk Protein**	ADM14327.1	minor ampullate spidroin [Uloborus diversus]	*Uloborus diversus*
**Minor Ampullate Silk Protein**	AFV31613.1	minor ampullate spidroin, partial [Araneus ventricosus]	*Araneus ventricosus*
**Minor Ampullate Silk Protein**	AFV31614.1	minor ampullate spidroin, partial [Araneus ventricosus]	*Araneus ventricosus*
**Minor Ampullate Silk Protein**	AFM29835.1	minor ampullate spidroin, partial [Argiope argentata]	*Argiope argentata*
**Minor Ampullate Silk Protein**	AFM29836.1	minor ampullate spidroin, partial [Argiope argentata]	*Argiope argentata*
**Minor Ampullate Silk Protein**	ABD61589.1	MiSp [Deinopis spinosa]	*Deinopis spinosa*
**Minor Ampullate Silk Protein**	ABD61597.1	MiSp [Uloborus diversus]	*Uloborus diversus*
**Minor Ampullate Silk Protein 1-Like**	ACB29694.1	minor ampullate spidroin 1-like protein [Latrodectus hesperus]	*Latrodectus hesperus*
**Minor Ampullate Silk Protein-Like**	ABR37276.1	minor ampullate spidroin-like protein [Nephilengys cruentata]	*Nephilengys cruentata*
**Minor Ampullate Silk Protein-Like**	ABR37277.1	minor ampullate spidroin-like protein [Nephilengys cruentata]	*Nephilengys cruentata*
**Minor Ampullate Silk Protein-Like**	ABR37278.1	minor ampullate spidroin-like protein, partial [Nephilengys cruentata]	*Nephilengys cruentata*
**Minor Ampullate Silk Protein-Like**	ADG57595.1	MiSp-like protein [Parawixia bistriata]	*Parawixia bistriata*
**Minor Ampullate Silk Protein-Putative**	AZAQ01030745.1	S.m._3 MiSp-putative	*Stegodyphus mimosarum*
**Minor Ampullate Spidroin 1**	ABC72645.1	minor ampullate fibroin 1 [Nephila antipodiana]	*Nephila antipodiana*
**Minor Ampullate Spidroin 1**	AAC14589.1	minor ampullate silk protein MiSp1 [Nephila clavipes]	*Nephila clavipes*
**Minor Ampullate Spidroin 1**	AF027735.1	minor ampullate silk protein MiSp1 [Nephila clavipes]	*Nephila clavipes*
**Minor Ampullate Spidroin 2**	AF027737.1	minor ampullate silk protein MiSp2 [Nephila clavipes]	*Nephila clavipes*
**Piriform Spidroin**	AEP25627.1	piriform spidroin [Araneus gemmoides]	*Araneus gemmoides*
**Piriform Spidroin**	ADN39425.1	piriform spidroin [Argiope trifasciata]	*Argiope trifasciata*
**Piriform Spidroin**	ADN39427.1	piriform spidroin [Nephila clavipes]	*Nephila clavipes*
**Piriform Spidroin**	ADN39426.1	piriform spidroin, partial [Nephila clavipes]	*Nephila clavipes*
**Piriform Spidroin-Like**	ADK56477.1	piriform-like spidroin [Nephilengys cruentata]	*Nephilengys cruentata*
**Piriform Spidroin-Putative**	AZAQ01087893.1	S.m._2 PiSp-putative	*Stegodyphus mimosarum*
**Pyriform Spidroin**	GGOF01074945.1	T.per_PySp_C_contig	*Tengella perfuga*
**Pyriform Spidroin**	GGRN01023505.1	8234_1_PySp_C	*Dolomedes triton*
**Pyriform Spidroin**	GGRN01007173.1	8231_1_D.tri_PySp_N	*Dolomedes triton*
**Pyriform Spidroin 1**	ACV41934.1	pyriform spidroin 1 [Latrodectus hesperus]	*Latrodectus hesperus*
**Pyriform Spidroin 1**	ADV40087.1	pyriform spidroin 1 [Latrodectus hesperus]	*Latrodectus hesperus*
**Pyriform Spidroin 2**	ADK92884.1	pyriform spidroin 2, partial [Nephila clavipes]	*Nephila clavipes*

**Silk Gland Protein 1**	AAR21194.1	silk gland protein 1 [Argiope amoena]	*Argiope amoena*
**Spidroin**	GGOF01078382.1	Tpercomp24302_c0-1_Sp_N	*Tengella perfuga*
**Spidroin**	GGRN01011518.1	7528_1_Sp_N	*Dolomedes triton*
**Spidroin 1**	AZAQ01020238.1	S.m.Sp1	*Stegodyphus mimosarum*
**Spidroin 1**	AAC38957.1	spidroin 1 [Nephila clavipes]	*Nephila clavipes*
**Spidroin 1a**	ACF71407.1	spidroin 1a [Avicularia juruensis]	*Avicularia juruensis*
**Spidroin 1b**	ACF71408.1	spidroin 1b, partial [Avicularia juruensis]	*Avicularia juruensis*
**Spidroin 1c**	ACF71409.1	spidroin 1c [Avicularia juruensis]	*Avicularia juruensis*
**Spidroin 2**	ACF71410.1	spidroin 2 [Avicularia juruensis]	*Avicularia juruensis*
**Spidroin 2**	AAC04503.1	spidroin 2, partial [Araneus bicentenarius]	*Araneus bicentenarius*
**Spidroin 2a**	AZAQ01082375.1	S.m.Sp2a	*Stegodyphus mimosarum*
**Spidroin 2b**	AZAQ01067030.1	S.m.Sp2b	*Stegodyphus mimosarum*
**Spidroin 2c**	AZAQ01099004.1	S.m.Sp2c	*Stegodyphus mimosarum*
**Tubuliform Spidroin**	AAX45292.1	tubuliform spidroin [Argiope aurantia]	*Argiope aurantia*
**Tubuliform Spidroin**	AAX45293.1	tubuliform spidroin, partial [Araneus gemmoides]	*Araneus gemmoides*
**Tubuliform Spidroin**	AAX45294.1	tubuliform spidroin, partial [Araneus gemmoides]	*Araneus gemmoides*
**Tubuliform Spidroin**	AAX45291.1	tubuliform spidroin, partial [Argiope aurantia]	*Argiope aurantia*
**Tubuliform Spidroin**	AAX45295.1	tubuliform spidroin, partial [Nephila clavipes]	*Nephila clavipes*
**Tubuliform Spidroin-1**	ADM14323.1	tubuliform spidroin 1 [Agelenopsis aperta]	*Agelenopsis aperta*
**Tubuliform Spidroin-1**	ADM14330.1	tubuliform spidroin 1 [Agelenopsis aperta]	*Agelenopsis aperta*
**Tubuliform Spidroin-1**	ADM14331.1	tubuliform spidroin 1 [Agelenopsis aperta]	*Agelenopsis aperta*
**Tubuliform Spidroin-1**	AAY28932.1	tubuliform spidroin 1 [Argiope argentata]	*Argiope argentata*
**Tubuliform Spidroin-1**	AAY28945.1	tubuliform spidroin 1 [Argiope argentata]	*Argiope argentata*
**Tubuliform Spidroin-1**	AAY28952.1	tubuliform spidroin 1 [Argiope argentata]	*Argiope argentata*
**Tubuliform Spidroin-1**	ADM14332.1	tubuliform spidroin 1 [Argiope argentata]	*Argiope argentata*
**Tubuliform Spidroin-1**	ADM14333.1	tubuliform spidroin 1 [Argiope argentata]	*Argiope argentata*
**Tubuliform Spidroin-1**	AAY28942.1	tubuliform spidroin 1 [Argiope aurantia]	*Argiope aurantia*
**Tubuliform Spidroin-1**	AAY28953.1	tubuliform spidroin 1 [Argiope aurantia]	*Argiope aurantia*
**Tubuliform Spidroin-1**	AAY28944.1	tubuliform spidroin 1 [Cyrtophora moluccensis]	*Cyrtophora moluccensis*
**Tubuliform Spidroin-1**	AAY28934.1	tubuliform spidroin 1 [Deinopis spinosa]	*Deinopis spinosa*
**Tubuliform Spidroin-1**	AAY28943.1	tubuliform spidroin 1 [Gea heptagon]	*Gea heptagon*
**Tubuliform Spidroin-1**	AAY28954.1	tubuliform spidroin 1 [Gea heptagon]	*Gea heptagon*
**Tubuliform Spidroin-1**	AAY28940.1	tubuliform spidroin 1 [Latrodectus geometricus]	*Latrodectus geometricus*
**Tubuliform Spidroin-1**	AAY28950.1	tubuliform spidroin 1 [Latrodectus geometricus]	*Latrodectus geometricus*
**Tubuliform Spidroin-1**	AAY28941.1	tubuliform spidroin 1 [Latrodectus hasseltii]	*Latrodectus hasseltii*

**Tubuliform Spidroin-1**	AAY28949.1	tubuliform spidroin 1 [Latrodectus hasseltii]	*Latrodectus hasseltii*
**Tubuliform Spidroin-1**	AAY28931.1	tubuliform spidroin 1 [Latrodectus hesperus]	*Latrodectus hesperus*
**Tubuliform Spidroin-1**	AAY28937.1	tubuliform spidroin 1 [Latrodectus hesperus]	*Latrodectus hesperus*
**Tubuliform Spidroin-1**	AAY28947.1	tubuliform spidroin 1 [Latrodectus hesperus]	*Latrodectus hesperus*
**Tubuliform Spidroin-1**	ADV40185.1	tubuliform spidroin 1 [Latrodectus hesperus]	*Latrodectus hesperus*
**Tubuliform Spidroin-1**	AAY28938.1	tubuliform spidroin 1 [Latrodectus mactans]	*Latrodectus mactans*
**Tubuliform Spidroin-1**	AAY28946.1	tubuliform spidroin 1 [Latrodectus mactans]	*Latrodectus mactans*
**Tubuliform Spidroin-1**	AAY28939.1	tubuliform spidroin 1 [Latrodectus tredecimguttatus]	*Latrodectus tredecimguttatus*
**Tubuliform Spidroin-1**	AAY28948.1	tubuliform spidroin 1 [Latrodectus tredecimguttatus]	*Latrodectus tredecimguttatus*
**Tubuliform Spidroin-1**	AAY28951.1	tubuliform spidroin 1 [Steatoda grossa]	*Steatoda grossa*
**Tubuliform Spidroin-1**	AAY28933.1	tubuliform spidroin 1 [Uloborus diversus]	*Uloborus diversus*
**Tubuliform Spidroin-1**	AFA43480.1	tubuliform spidroin 1, partial [Argiope amoena]	*Argiope amoena*
**Tubuliform Spidroin-1**	ABD24296.1	tubuliform spidroin 1, partial [Latrodectus hesperus]	*Latrodectus hesperus*
**Tubuliform Spidroin-1**	AAY90151.1	TuSp1 [Nephila antipodiana]	*Nephila antipodiana*
**Tubuliform Spidroin-Like**	ABR37274.1	tubuliform spidroin-like protein [Nephilengys cruentata]	*Nephilengys cruentata*
**Tubuliform Spidroin-Putative**	AZAQ01117603.1	S.m._4 TuSp-putative Tubuliform spidroin putative	*Stegodyphus mimosarum*
**Tubulliform Spidroin**	GGOF01102549.1	T.per_TuSp_C_contig	*Tengella perfuga*
**Tubulliform Spidroin**	GGOF01015106.1	Tpercomp127803_c1-1_TuSp_N	*Tengella perfuga*
**Tubulliform Spidroin**	GGRN01041094.1	D.tri_TuSp_C_contig	*Dolomedes triton*
**Tubulliform Spidroin**	GGRN01011637.1	5425_1_D.tri_TuSp_N	*Dolomedes triton*

Table S5. Spidroin sequences included in phylogenetic analyses

Spidroin Name	Species	N-terminal region GenBank Accession	C-terminal region GenBank Accession
***A.ape*_MaSp**	*Agelenopsis aperta*	HM752573	AAT08436
***A.ape*_TuSp1**	*Agelenopsis aperta*	HM752576	--
***A.arg*_AcSp**	*Argiope argentata*	AHK09813	AHK09813
***A.arg*_Flag**	*Argiope argentata*	--	MF955778
***A.arg*_MaSp1**	*Argiope argentata*	AWK58623	AWK58705
***A.arg*_MaSp2**	*Argiope argentata*	AWK58645	AWK58747
***A.arg*_MaSp3**	*Argiope argentata*	AWK58729	AWK58636
***A.arg*_MiSp**	*Argiope argentata*	AWK58671	AWK58662
***A.arg*_PySp1**	*Argiope argentata*	AQR58363	AQR58363
***A.arg*_TuSp1**	*Argiope argentata*	ATW75951	ATW75951
***A.dia*_AcSp**	*Araneus diadematus*	AWK58687	AWK58698
***A.dia*_Flag**	*Araneus diadematus*	AWK58733	AWK58723
***A.dia*_Masp1**	*Araneus diadematus*	AWK58624	AWK58706
***A.dia*_MaSp2**	*Araneus diadematus*	AWK58648	AWK58752
***A.dia*_MaSp3**	*Araneus diadematus*	--	AWK58637
***A.dia*_MiSp**	*Araneus diadematus*	--	AWK58663
***A.dia*_PySp1**	*Araneus diadematus*	AWK58658	AWK58716
***A.dia*_TuSp1**	*Araneus diadematus*	AWK58641	AWK58742
***B.cal*_fibroin1**	*Bothriocyrtum californicum*	HM752562	EU117162
***D.ten*_fib1**	*Dolomedes tenebrosus*	--	AF350269
***D.ten*_fib2**	*Dolomedes tenebrosus*	--	AF350270
***L.hes*_AcSp1**	*Latrodectus hesperus*	AFX83557	AFX83557
L.hes _Flag	*Latrodectus hesperus*	AWK58736	AWK58725
***L.hes* _MaSp1**	*Latrodectus hesperus*	F595246	F595246
***L.hes* _MaSp2**	*Latrodectus hesperus*	F595245	F595245
***L.hes* _MaSp3**	*Latrodectus hesperus*	AWK58730	AWK58638
***L.hes* _MiSp**	*Latrodectus hesperus*	ARA91152	ARA91152
***L.hes* _PySp1**	*Latrodectus hesperus*	AWK58659	AWK58717
***L.hes* _TuSp1**	*Latrodectus hesperus*	AWK58642	AWK58744
***N.cla*_AcSp1**	*Nephila clavipes*	AWK58691	AWK58702
***N.cla*_Flag**	*Nephila clavipes*	AWK58737	AWK58726
***N.cla*_MaSp1**	*Nephila clavipes*	AWK58628	AWK58709
***N.cla*_MaSp2**	*Nephila clavipes*	AWK58653	AWK58758
***N.cla*_MiSp**	*Nephila clavipes*	AWK58679	AWK58667
***N.cla*_PySp1**	*Nephila clavipes*	AWK58660	AWK58718
***N.cla*_TuSp1**	*Nephila clavipes*	AWK58643	AWK58745
***N.cla*_TuSp1**	*Nephila clavipes*	AWK58643	AWK58745
***S.mim*_AcSp-putative**	*Stegodyphus mimosarum*	KFM79920	KFM79920
***S.mim*_MaSp-putative-a**	*Stegodyphus mimosarum*	--	KFM83271
***S.mim*_MaSp-putative-c**	*Stegodyphus mimosarum*	--	JT038023
***S.mim*_MaSp-putative-d**	*Stegodyphus mimosarum*	KFM59474	KFM59474
***S.mim*_MaSp-putative-e**	*Stegodyphus mimosarum*	KFM74936	--
***S.mim*_MaSp-putative-f**	*Stegodyphus mimosarum*	KFM61798	--
***S.mim*_MaSp-putative-g**	*Stegodyphus mimosarum*	KFM57717	--
***S.mim*_MaSp-putative-h**	*Stegodyphus mimosarum*	KFM61802	KFM61800
***S.mim*_MaSp-putative-i**	*Stegodyphus mimosarum*	KFM79313	KFM79313
***S.mim*_Misp-putative**	*Stegodyphus mimosarum*	KFM62627	KFM62627
***S.mim*_PiSp-putative**	*Stegodyphus mimosarum*	KFM75168	KFM68615
***S.mim*_Sp1**	*Stegodyphus mimosarum*	--	KFM60634
***S.mim*_Sp2a**	*Stegodyphus mimosarum*	KFM73910	--
***S.mim*_Sp2b**	*Stegodyphus mimosarum*	KFM70693	--
***S.mim*_TuSp-putative**	*Stegodyphus mimosarum*	KFM79920	KFM79920
